# Edges and gradients in lightness illusions: Role of optical veiling glare

**DOI:** 10.3389/fpsyg.2022.958787

**Published:** 2022-12-15

**Authors:** John J. McCann, Vassilios Vonikakis, Alessandro Rizzi

**Affiliations:** ^1^McCann Imaging, Arlington, MA, United States; ^2^Advanced Digital Sciences Center, Singapore, Singapore; ^3^Dipartimento di Informatica, Università degli Studi di Milano, Milan, Italy

**Keywords:** lightness illusions, retinal glare, visibility of glare, scene content, HDR and LDR scenes, python code-retinal contrast, glare’s paradox, neural spatial processing

## Abstract

Lightness Illusions (Contrast, Assimilation, and Natural Scenes with Edges and Gradients) show that appearances do not correlate with the light sent from the scene to the eye. Lightness Illusions begin with a control experiment that includes two identical Gray Regions-Of-Interest(GrayROI) that have equal appearances in uniform surrounds. The Illusion experiment modifies “the-rest-of-the-scene” to make these GrayROIs appear different from each other. Our visual system performs complex-spatial transformations of scene-luminance patterns using two independent spatial mechanisms: optical and neural. First, optical veiling glare transforms scene luminances into a different light pattern on receptors, called retinal contrasts. This article provides a new Python program that calculates retinal contrast. Equal scene luminances become unequal retinal contrasts. Uniform scene segments become nonuniform retinal gradients; darker regions acquire substantial scattered light; and the retinal range-of-light changes. The glare on each receptor is the sum of the individual contributions from every other scene segment. Glare responds to the content of the entire scene. Glare is a *scene-dependent* optical transformation. Lightness Illusions are intended to demonstrate how our “brain sees” using simple-uniform patterns. However, the after-glare pattern of light on receptors is a morass of high-and low-slope gradients. Quantitative measurements, and pseudocolor renderings are needed to appreciate the magnitude, and spatial patterns of glare. Glare’s gradients are invisible when you inspect them. Illusions are generated by neural responses from “the-rest-of-the-scene.” The neural network input is the simultaneous array of all receptors’ responses. Neural processing performs vision’s second *scene-dependent* spatial transformation. Neural processing generates appearances in Illusions and Natural Scenes. “Glare’s Paradox” is that glare adds more re-distributed light to GrayROIs that appear darker, and less light to those that appear lighter. This article describes nine experiments in which neural-spatial-image processing overcompensates the effects of glare. This article studies the first-step in imaging: s*cene-dependent* glare. Despite near invisibility, glare modifies all quantitative measurements of images. This article reveals glare’s modification of input data used in quantitative image analysis and models of vision, as well as visual image-quality metrics. Glare redefines the challenges in modeling Lightness Illusions. Neural spatial processing is more powerful than we realized.

## Introduction

Vision, and Images made for humans, have three major stepping stones: light from the scene, receptors’ response to light, and appearances. This article studies Lightness Illusions, glare, and the visual pathway that leads to appearances. Optical Veiling Glare is the first step in all of imaging with lenses. It is the first spatial transformation of scene luminance information. Glare modifies the pattern of light falling on retinal and cameras’ receptors. Glare redistributes light from high-luminance scene segments into low-luminance regions. The amount of received glare from a single scene element, or single donor pixel is tiny. However, glare is the sum of all the millions of tiny contributions from all other scene pixels. Glare makes a unique (scene-dependent) light contribution to all scene pixels (McCann and Rizzi, 2011; McCann et al., 2018).

In a 1,000,000 pixel image, the glare added to each individual pixel is the sum of glare contributions from 999,999 other pixels. That process is repeated a million times to calculate the retinal image. In computationally efficient FFT convolutions there are the equivalent of 10^12^ glare contributions. Glare requires a *scene-dependent model*. All input scene pixels are necessary to calculate each *scene-dependent* pixel’s output.

The science of Imaging uses two different quantitative metrics. First, optics uses the International System of Units (SI), made up of 7 base units (second, meter, kilogram, ampere, etc.) For visible light SI-7 includes the candela (cd), and derived-unit luminance [candela/per square meter; [National Institute of Standards and Technology (NIST), 2022]. This standard is traceable to human detection thresholds of light, and is based on wavelength and the energy of photons. It quantifies the energy required for specific human Light/Matter minimum detection thresholds at atomic and molecular levels. Here, experimenters ask the observers, did you detect light. Their answer reports the amount of light at threshold, and its calibration reports *quanta catch* (Hecht et al., 1942). This is vision’s *scene-independent* measurement.

Some theories, and practical technologies use *scene-independent models*. They use only a single scene pixel’s *quanta catch* to calculate each pixel’s final signal. *Scene-independent* models assume that the *quanta catch* of each individual pixel is all the information from the scene that is necessary to model the response function to light in all pixels, and in all images. For example, silver-halide film responses are accurately modeled by the *quanta catch* of microscopic regions of film. The film has a fixed-response function to light. Every scene segment with constant light stimulus generates identical film optical densities independent of the “rest of the scene.” (The film is *scene-independent,* however camera bodies and lenses introduce glare (Jones and Condit, 1941), making cameras *scene-dependent*.) Other examples of *scene-independent* models are: CIE-Colorimetry, CIE Color Appearance Models (CIECAM), most digital cameras and displays. These calculations allow only single pixel scene radiance inputs from the scene to predict single-pixel quanta response. Scenes with millions of pixels requires millions of independent calculations. Digital *scene-independent* calculations, use hardware, firmware, and Look-Up-Tables (LUTs) in pipelines for efficiency, but they are unresponsive to optical glare, and all of human vision’s *scene-dependent* mechanisms.

Practical Imaging technology and Image Quality use *appearance* metrics to evaluate human response to prints and displays. It measures response at the opposite end of the human visual pathway from *quanta catch*. Instead of quantifying local molecular events, it measures vision’s spatial-image processing of all 100 million receptor outputs. Here, experimenters ask observers which color or lightness sample in a standard collection does the ROI match. Their answer reports *appearances* that are *scene-dependent*.

Psychophysics has innumerable examples of [*appearance* ≠ *quanta catch*]. Color Constancy (McCann, 2021d) and Lightness Illusions demonstrate that successful models of vision require input data from “the-rest-of-the-scene.” Since the 1950’s neuroanatomy, neurophysiology, and psychophysics have documented that the human visual pathway is a cascade of spatial comparisons. Retinal receptors, amacrine, horizontal, ganglion, ipRGC, lateral geniculate, striate cortex, blobs, and v4 cells perform different types of spatial comparisons at different spatial resolutions and orientations (Hubel and Wiesel, 1965; Oyster, 1999).

Retinal receptors’ outputs are not relayed as independent pixel responses to the brain. They become time-modulated, spatial comparisons that apply different image-processing mechanisms at every stage. The input data for vision require all receptor responses simultaneously to perform all of its analysis. Vision models requires efficient spatial image processing of all pixels to calculate appearances. The interactions of all spatial scene elements generates appearance (McCann and Rizzi, 2011:pp. 173–375).

This article studies how glare affects normal-dynamic-range Lightness Illusions for two reasons. First, Lightness Illusions demonstrate that vision is the result of *scene-dependent* spatial processing. Second, these Illusions work well in the limited range of light found on normal low-dynamic-range displays. Lightness Illusions contain two identical scene-luminance segments that are identified as the “regions-of-interest” (ROI). Those segments appear identical if the “rest-of-the-scene” is restricted to a single uniform luminance. However, the designers of Illusions introduce clever “rest-of-the-scenes” that makes two identical ROI luminances have different *appearances* in the same scene. Since glare redistributes light from all of the scene’s pixels, the question becomes how does the Illusion’s “rest-of-the-scene” alter those equal scene-luminance segments. Glare has its strongest effects on the darkest scene segments, moderate effects on mid-range segments; and minimal effect on the brightest regions. However, glare’s most influential effects are found at edges between different scene segments, and changes in uniformity.

High-Dynamic-Range (HDR) studies (McCann and Rizzi, 2011) have renewed interest in glare’s effect on appearance pioneered by Hering (in Hurvich and Jameson, 1966) and Fry and Alpern (1953) and Fry and Alpern (1954). Vos et al. (1976) measured the eye’s Glare Spread Function (GSF), and Vos and van den Berg’s (1999) standardized the newer CIE GSF; expanded by Franssen and Coppens (2007). McCann and Vonikakis (2018), expanded Rizzi/Farup’s MATLAB® program for converting all scene luminances to retinal light levels. The present submission introduces Python (open-source code) that performs the same calculations. Both programs analyze the actual spatial distribution of light on receptors.

The Gregory and Gombrich (1980) review of illusions includes all types of identical stimuli that are modified by the rest of the scene (lines, constant-size objects, and constant light stimuli). All illusions appear markedly different because of the influence of the “rest-of-the-scene.” Observing ROI’s different appearances, in Lightness Illusions and their controls, side-by-side, is compelling evidence of vision’s scene-dependent spatial processing. There are three Lightness Illusion types: Simultaneous Contrast, Assimilation, and Edge/Gradient scenes [Edwin Land’s Black and White Mondrian Land and McCann (1971), and Adelson’s (1995)]. All have equal-luminance pairs of scene segments(ROI) that appear different because of the influence of “the-rest-of-the-scene.” Many visual properties could contribute to Lightness appearances: adaptation, lateral-neural interactions, multi-resolution processing, edges and gradients, perceptual frameworks. This article adds scene-dependent optical veiling glare to this list of appearance mechanisms affecting Lightness Illusions.

In order to study human vision, we need to understand the sequence of events along the visual pathway. Each stage has a unique input/output response function to light:

- Stage 1. Light from scenes (*scene luminance*: measured with photometer)
- Stage 2. Light on the retina (*retinal contrast*: after optical veiling glare)
- Stage 3. Light/Matter interactions (linear sums of rod and cone quanta catch)
- Stage 4. Receptor output ➜ Neural input (log quanta catch)
- Stage 5. Image processing in the visual pathway (Neural-Spatial comparisons)
- Stage 6. Appearance (Psychophysical Appearance and Perception data)

There is universal agreement about the facts listed in the first four stages: (1) Scenes are described as arrays of all calibrated *scene luminances* (cd/m^2^), each at a calibrated visual angle; (2) The pattern of light on the retina equals scene convolved with the standard CIE Glare Spread Function (GSF); (3) Light/Matter biochemical reactions, initiated by photons, takes place at a molecular level within cubic microns (linear sum of rod and cone quanta catch); (4) Receptor’s chemical output (at receptor’s neural junctions at the other end of the cell) generates a response function equal to log quanta catch response across its synapse in the horizontal cells (Hartline and Graham, 1932; Werblin and Dowling, 1969; Oyster, 1999).

In summary, the sequence of different human Response Functions to light is:

1. Scene luminance = cd/m^2^
2. Glare redistributes light
3. Visual pigments count photons = linear quanta catch
4. Receptor output ~ log quanta catch

The physiology of receptors presents a compelling case that receptor response is proportional to log quanta catch for a spot of light on a receptors.

Psychophysical research on Uniform Color Spaces shows a different total Response Function to Light in Stage 6. Munsell asked observers to make judgments of uniform distances in Lightness, Hue and Chroma. This data established a Colorimetric Uniform Space describing appearances in complex scenes (Newhall et al., 1943). Munsell’s Lightness is proportional to the cube-root of luminance. Many experiments have verified Munsell’s results. CIE(L*) has a cube-root *response function* to scene luminances (Wyszecki and Stiles, 1982; McCann and Rizzi, 2008).

The analysis of Scene Content, *scene-independent*, and *scene-dependent* experiments are key to understanding the apparent conflict between physiology and psychophysics. Physiology experiments measure receptor cells in a dark room with a small spot of light on them. These are *scene-independent* experiments. Psychophysical Uniform Lightness Scale experiments are performed in a light environment as a part of a complex scene. These are *scene-dependent* experiments. The physiological experiment had minimal glare, while the psychophysical experiments had considerable glare.

Stiehl et al. (1983) made an HDR Lightness Scene composed of neutral density filters whose appearances are equally-spaced Lightness patches in a uniform surround. They measured the luminances of each of the equally-spaced Lightness steps. They plotted those luminances vs. Lightness step and found the cube-root function often reported in the literature. This complex scene contained nine Lightness segments that observers selected to be equal steps in Lightness. The high-luminance surround around each segment added glare to each of them. The cube-root plot of the scene before glare means, when starting from Max luminance, the difference in log luminance between each Lightness step increases with every darker step. That is, the scene’s log-luminance difference between max and the next darker Lightness is the smallest value; and the scene’s log-luminance difference gets larger with every darker Lightness step.

Stiehl calculated the *retinal contrast* of these equally-spaced Lightness using the Vos et al. (1976) GSF. This data showed that glare added variable amounts of stray light to each of the equally spaced Lightness segments. The plot retinal contrast vs. log luminance was fit by a straight line. That means that all of the sequence of equally spaced Lightness segments had a constant difference in log luminance on the retina. The calculated glare added the amount of stray light needed to make all decrements appear equal.

Another way to look at this result is that the observers had to decrease the luminance of darker patches to make the Lightness steps equal. The darker the step, the greater the decrease needed.

Uniform Color Space target scenes have considerable glare. Observers reported that equally-spaced Lightnesses have equal decrements in log luminance. The sum of [scene luminance + glare] equals constant log-luminance decrements. The assumption of zero glare generates the cube-root Lightness function in CIE(L*). Calculating the light on the retina generates the straight line log-luminance function. Physiological receptor response is a log-luminance function. Lightness is promotional to receptor response in these high-glare scenes.

Our visual system performs complicated spatial transformations of light patterns from scenes. Measurements of appearances in HDR scenes (McCann and Rizzi, 2007, 2009, 2021a, 2021e; Rizzi and McCann, 2009; McCann and Vonikakis, 2018) showed large reductions of retinal-dynamic range in maximal-glare scenes. Two transparent films were superimposed to make 40 patches (white-to-black) with scene luminance range of 5.4 log units. All patches were surrounded by a max-luminance surround. After intraocular glare the retinal contrast range was 1.5 log units. In a nearly million:1 range scene, glare reduced the range of light on the retina to 33:1. The scene’s appearance varied from bright white to very-dark black.

A second experiment changed the background around each of the 40 patches from max-luminance to min-luminance. In this nearly million:1 range scene, glare reduced the range of light on the retina to 5,000:1. The second scene’s appearance varied from bright white to very-dark black. Observers reported that whites appeared the same white in both experiments. Remarkably, blacks appeared the same black in both experiments despite the change in range from 33:1 to 5,000:1. Appearances over the range of white to black have variable *scene-dependent* response functions to light on receptors (McCann and Vonikakis, 2018). In all cases, these response functions are all straight-line log luminance plots, with variable, scene-dependent slopes (Stiehl et al., 1983; McCann and Vonikakis, 2018).

This previous HDR glare study described an open-source computer program code using MATLAB programming language. The present study describes a new more accessible version using Python (open-source) programming language. Both programs describe techniques to compare the calibrated image of *scene luminances* with the calculated *retinal contrast* image. A computational model of *appearances* must first calculate the light imaged on the retina. This article describes computer calculations, based on the CIE Standard for Intraocular Glare (Vos and van den Berg, 1999), which makes specific adjustments for observer’s, age and color of iris. Our new software is implemented in Python. Both code and programming language are freely available to all researchers. (The code is in Data Sheet 1 in Supplementary material.)

**Figure 1:**
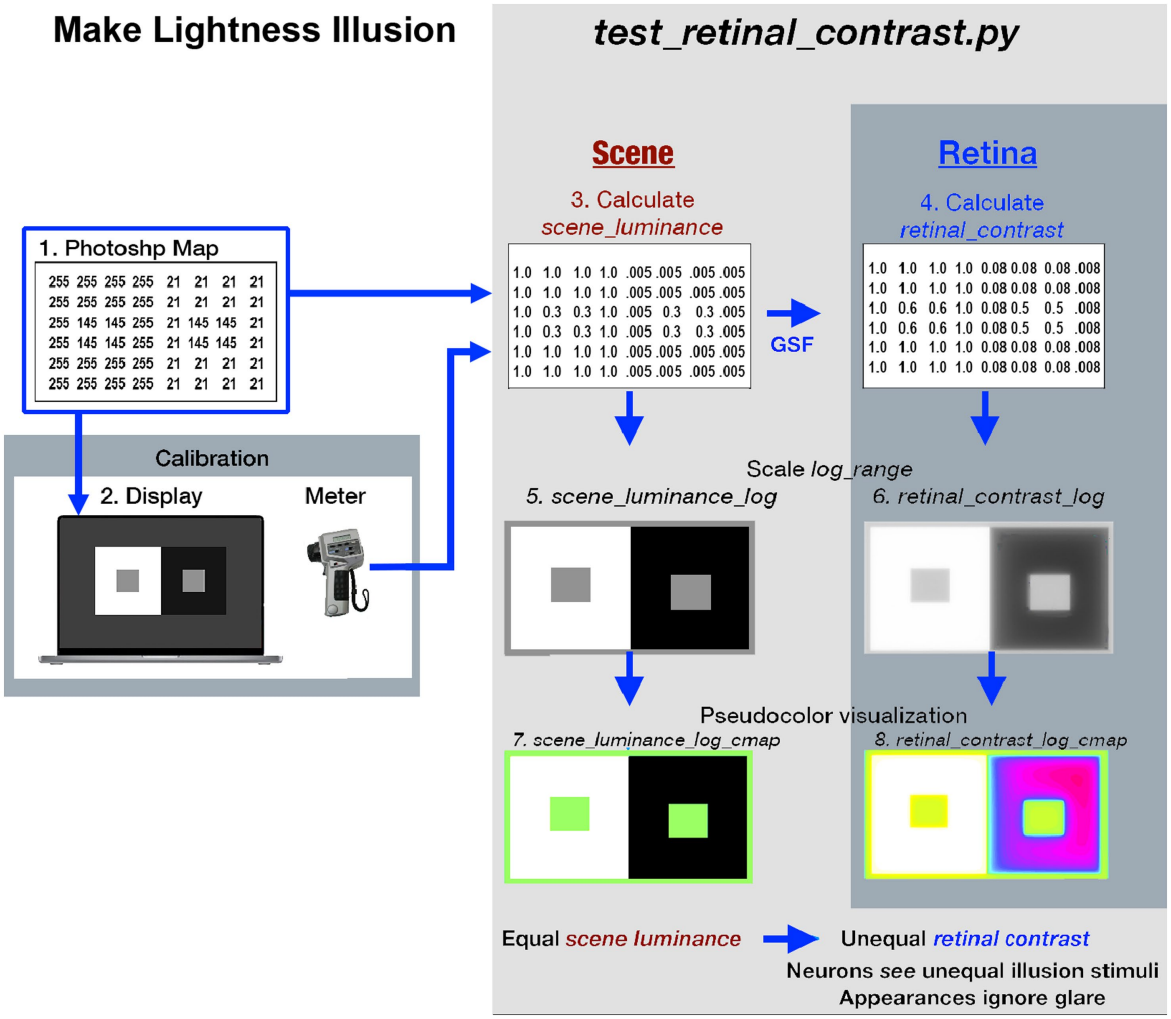
Illustrates the eight different images used in the Lightness Illusion’s construction, calibration of scene luminance input, and retinal contrast calculation of the light falling on receptors, followed by the analysis of the effects of glare. The image (1) is the Photoshop® digital file (the array of 8-bit values) of a Contrast Illusion. Contrast has two Gray Regions-of-Interest (ROI), surrounded by max digit on the left, and min digit on the right. The image (2) is that 8-bit array displayed on the Apple XDR powerbook screen. Using a Konica Minolta C100A telephotometer, the experimenters measured the scene luminances of light emitted by the screen at all digital inputs. Using this calibration, max-White was set to digit 255; the min-Black to digit 21, so that the range of measured luminances of the display was 200:1 [log_range = 2.3]. The experimenters adjusted the digital values of the GrayROIs to be equal, and to optimize the Contrast Illusion’s effects on Grays’ appearances. The image(3) made by the Python program, is a digital file that uses photometer measurements, and Photoshop’s map to make the <*scene_luminance >* (64-bit per pixel double precision floating point) file. This file is the Scene that is convolved with the CIE GSF to calculate <*retinal_contrast >* of the pattern of light on the Retina (image 4). These 64-bit double precision arrays, images (3) and (4), cannot be accurately rendered on a display at full precision. The next two rows show the four images used to analyze and visualize the effects of glare. Images (5) and (6) are converted from 64-bit double precision data to 8-bit log, scaled to the Scene’s [log_range = 2.3]. These images are used for numerical analysis of pixels’ values, and their plots of *Scene* and Retina. The bottom-row uses Pseudocolor renditions to visualize the spatial distribution of light on the retina. Many glare-generated gradients in retinal contrast are invisible in <*grayscale*>. Pseudocolor rendering makes the spatial patterns of these gradients highly visible. Each Lightness Illusion uses these 8 different images to create the Illusion; calibrate its Scene luminances; calculate the light on the Retina; and quantitatively analyze glare’s re-distribution of light.

Luminance, unambiguously defined in physics, is the measured input array used by the Glare Spread Function (GSF) convolution in the Python program. This article defines *retinal contrast* as the name of the program’s first calculated output image. The GSF convolution conserves the total energy in the input *scene_luminance* array. It redistributes all of the input energy into the output image. As described by Hecht et al. (1942) the light falling on receptors is attenuated by front surface reflection, intraocular and macular pigment absorptions. The eyes’ pupil size, and pre-retinal light absorptions are not accounted for in our program. This article uses *retinal contrast* as the specific term for the amount of light imaged on the retina. It is the normalized, linear photopic energy per pixel in a flat array congruent with the flat visual test targets. We do not use the term retinal luminance because our calculation does not measure intraocular light attenuation. *Retinal contrast* is the convolution’s output (normalized pattern of light on receptors).

(Figure 1 **-**left-side) illustrates the fabrication and calibration of each Lightness Illusion. The <*test_retinal_contrast.py >* program (right-side) converts the Illusion’s Photoshop map using calibration measurements of each digit values to make the <*scene_luminance* > input array. The program calculates <*retinal_contrast>*, and provides tools to analyze the effects of glare.

In today’s world, most visual media are seen on electronic displays. Their ~10% surface reflectance appears black in displayed images. Digital displays of illusion have replaced those on printed pages. Investigating appearances in Natural Scenes have become the study of edges and gradients of light, replacing studies of printed reflectance and ambient illumination. It is difficult to discuss illusions on a screen in terms of its reflectance and its illumination. Its reflectance is irrelevant background light, because the image is all emitted light. Displays emit illumination with edges and gradients. The thoughtful explanation of illusions has moved on to the analysis of spatial patterns of light. The analysis of reflectance and illuminance becomes a historical footnote, while the scene luminances’ spatial array is the source of information that generates the array of receptor’s quanta catch, that generate appearances.

The appearance of every segment in illusions and Natural Scenes involves the entire human visual system. That system has a visual angle of 120°, and uses the simultaneous responses of all 100 million retinal receptors. Neural-spatial processing compares all the receptor responses to generate an illusion’s appearances. Glare simply adds a new layer of complexity to neural-spatial vision’s input from receptors. Receptors capture quanta, and neural-spatial comparisons find edges, sharpens them, and ignores the subtle gradients caused by glare. This article’s study of Lightness Illusions is limited to glare’s transformation of scene luminance inputs to all retinal contrast outputs, and the appearances of retinal contrasts. This article does not model, nor predict appearances of Lightness Illusion segments. The study of computational models of appearance is an enormous topic that involves many different approaches (Land and McCann, 1971; Frankle and McCann, 1983; Adelson, 2000; Gilchrist, 2006; McCann and Rizzi, 2011; Blakeslee and McCourt, 2015; McCourt et al., 2016; Rudd, 2020). This topic is far too large to fit in the scope of this paper.

This article simply presents Lightness Illusions, and asks the reader whether ROI A is lighter, the same, or darker than ROI B. It also asks if particular scene segments appears to be uniform. This study shows that glare is hard to see; namely its effects are nearly invisible, or invisible. Because it is so hard to appreciate glare by visual inspection, quantitative analysis of glare is required in evaluating models of vision, imaging, and particularly image-quality assessments.

Both Glare and Neural Spatial processing are scene-dependent mechanisms. While more efficient scene-independent calculations can model receptor quanta catch for spots of light in a no-light surround (Colorimetry), they cannot accurately calculate appearances in Natural Scenes (McCann, 2020). Glare is the first spatial transformation of scene information. Quantitative studies of human retinal images shows that neural spatial mechanisms can overcompensate for glare (McCann, Vonikakis, Rizzi,2018:pp.142–159). The study of neural processing requires quantitative data from all of its input, namely the array of all receptor responses.

Section Methods and materials: Calculating and analyzing intraocular glare of this article describes how to calculate retinal_contrast and how the program uses pseudocolor to visualize it. Section Results describes nine Lightness Illusions, their numerical analysis, and pseudocolor rendering. These results identify Glare’s Paradox, namely that human neural processing overcompensates glare’s effects in Contrast, but not in Assimilation. Section Discussion explores the visibility of gradients of light; compensation for glare by neural spatial processing; and glare’s role in Image Quality metrics.

## Methods and materials: Calculating and analyzing intraocular glare

As illustrated in Figure 1, we made an image in Photoshop® of the familiar Contrast Illusion (ROI-Grays darker in White; lighter in Black). We sent the illusion’s digital file to a calibrated display [range of cd/m2 set to 200:1]. We measured the luminance of all scene segments. The Python program that calculates glare’s effects on Illusions has two parts. First, it makes an array of calibrated display luminances and convolves it with the CIE GSF. Second, it makes meaningful visualizations of the millions of pixels in each scene, and its retinal image.

### Calculating retinal image

**Figure 2:**
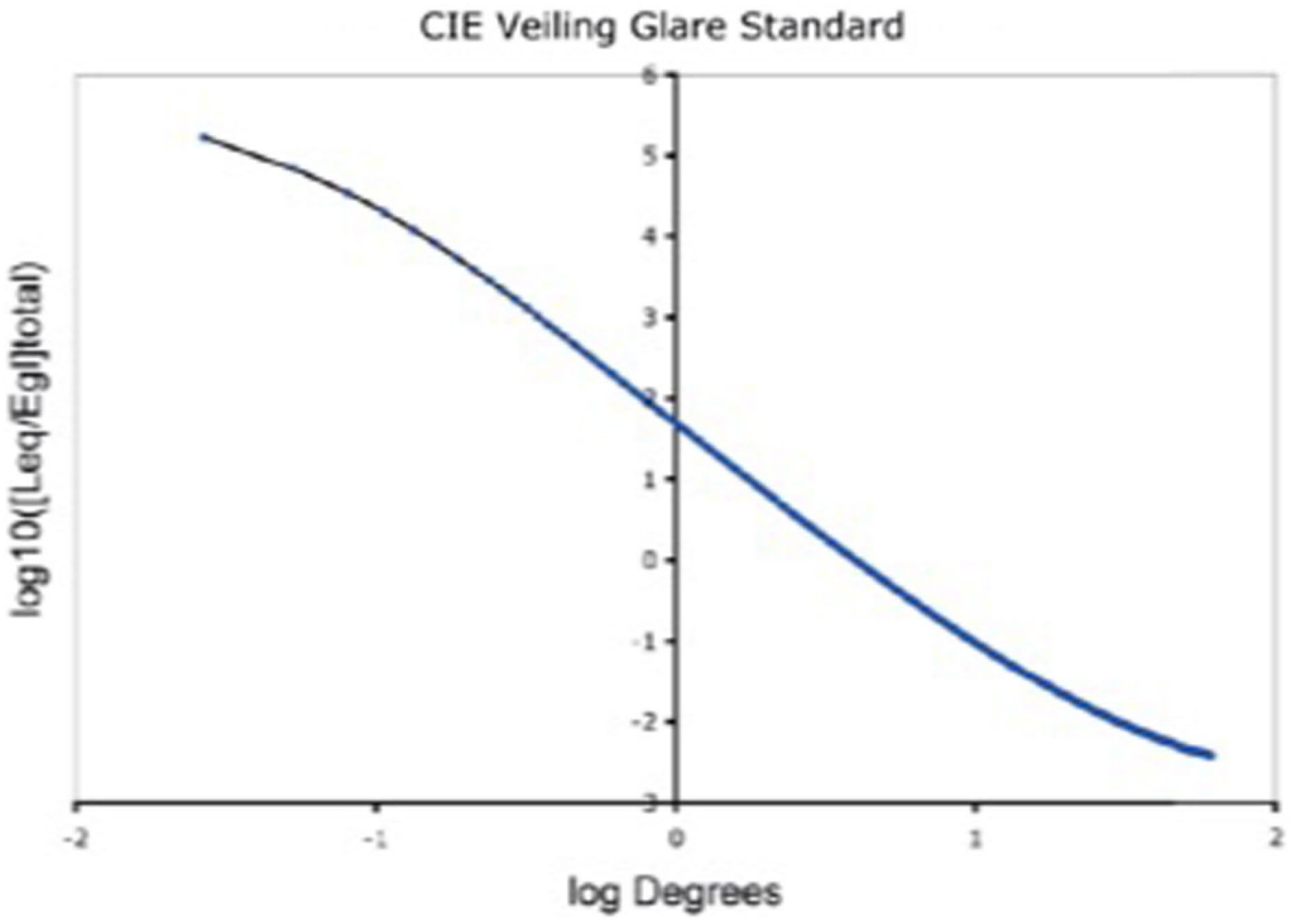
Glare Spread Function plotted on log–log axes. Note the extreme ranges of these axes. The horizontal *visual-angle* axis covers (1 min to 60°). The vertical axis plots the decrease in glare as the function of the angular separation between donor pixel and receiving pixels. It covers 8 log_10_ units (150,000 to 0.005). Despite its range, it does not approach a constant asymptote. The glare on each receiving pixel is the unique sum of contribution of all the other scene pixels. Glare is a scene-content-dependent transformation of scene luminances.

The GSF specifies the fraction of a pixel’s light scattered onto every other pixel in the whole scene. It varies as a function of angular distance (1/60° to 60°) between donor and receiving pixel. The convolution sums all the millions of glare contributions from all the other pixels. Hence, 64-bit floating-point double precision was used for the convolution. The retinal image calculation (Vos and van den Berg, 1999) covers 60° visual angle, and the range of scattered light [(log10 [Leq/Egl]total)] covers 8 log10 units (Figure 2).

### Optical glare spread function

The calculation of light on the retina used the GSF formula (Vos and van den Berg, 1999; Equation (8) formula) to calculate the spatial distribution of the light on the retina. The retinal image is the sum of scene luminance, plus light scattered into each pixel. The amount scattered into each pixel depends on the luminance of the donor pixel and its angular separation between the donor and receiving pixels. CIE GSF calculations are described in McCann and Vonikakis (2018) that contains additional background information. Using this CIE standard, we calculated the relative luminance at each pixel (Leq/ Egl). It is the ratio of Equivalent Veiling Luminance (Leq in cd/m2) and Glare Illuminance at the Eye. In the calculations we used brown eye color pigment = 0.5 and age = 25 to calculate predictions for young observers, with minimal-glare vision.

### Glare spread function convolution filter kernel

We first compute the 2D filter kernel (Vos and van den Berg, 1999; equation (2) CIE-GSF), which will be used in the convolution with <scene_luminance>. The kernel’s radius is equal to the maximum size of the luminance input array (+1 for symmetry). This ensures that every pixel will be able to “affect” all others during convolution. When the center of the kernel is positioned on the top-left pixel, the kernel should cover the whole luminance input array. The python code is written to process any size of input luminance array. We have to adjust the kernel size, to accommodate the input size, and maintain angular calibration of the image. Even though the radius of the kernel is large, its values are never zero. This means that every position in the retinal input array will contribute to all the others. Once the 2D filter kernel values are calculated from Equation (2), they are normalized by their total sum, ensuring that all add up to unity and thus, no energy is introduced during the convolution. Also, there is no radial distance at which the glare contribution reaches a constant asymptotic value.

The next operation computes the retinal image by convolving the filter kernel on the scene luminance array, resulting in retinal contrast. Performing the convolution, with such a large size kernel in the spatial domain, is computationally expensive, since each of N pixels is affected by all others. As such, the complexity of this operation is O(N2). Performing the convolution in the frequency domain shortens computation time, resulting in O(NlogN) complexity. Our Python code performed MATLAB’s < imfilter>, convolution in the frequency domain using the Fast Fourier Transform (FFT).

The calculation of the 2D filter kernel, as well as the convolution operation with the <scene_luminance> input array, are implemented in <test_retinal_contrast.py > (see Python script in Github repository (Vonikakis, 2022).

### Input/output ranges

The calculation of retinal contrast from scene luminance modifies an image’s dynamic range. There are three aspects to managing range:

- First-Glare redistributes a very small fraction of light from all pixels to all other pixels
  - largest sources of glare light are the highest luminance pixels
  - largest recipients of light are the lowest luminance pixels
  - input image must represent both the entire range of *scene_luminances,* and tiny glare contributions accurately.
- Second-Computational precision of pixel values. The GSF convolution uses linear, 64-bit double floating point precision to calculate the result of all pixels’ contributions, and the accumulation of these tiny amounts of light. This need for precision includes the padding of external input boundaries in the convolution.
- Third-Visualization of input/output information. Calibrated images can exceed display’s range used to visually inspect them. Displayed rendition of (in/out) calculation data must account for display’s firmware luminance transformations of digit values, and vision’s response to light. We also need tools to visually inspect scenes that exceed the display’s range. We need to inspect data in *gradients-in-luminance* by making them visible using pseudocolor.

### Computational padding

Computation of glare values near borders of the input array requires special treatment, because part of the kernel goes out of the area of the input array. In our Python code, we used a “boundary replication” padding approach, similar to the MATLAB “replicate” option for the imfilter function. According to this, the pixels of the outer rim of the image are replicated in order to cover the padded area.

- If all the outer edge pixels in <*map.tif >* file are White(max-digit), the”boundary replication” becomes the equivalent of a uniform white surround 9 times the area of <*map.tif>*, with the map placed at the center. Consequently, glare is calculated as if the target was on a uniform white surround.
- If the outer edges are min-luminance, glare is calculated as if the target is in a darkroom on a black background.

Vos and van den Berg (1999) describe the shape of the GSF. That shape does not include the glare loss of (re-distributed) light from every pixel. In our program the filter kernel is normalized so the sum of all output retinal_contrast equals the sum of all input scene luminances. In the <test_retinal_contrast.py > program we verified the kernel in each calculation, e.g., [kernel sum = 0.999999999999998] was a typical result. Without this normalization step, the sum of output could exceed the sum of input. The filter calculates the light distribution projected on a sphere (CIE GSF); and the program converts that to the light projected on a plane. Input pixels and output pixels are planar and have identical dimensions. It does not include the effects of pre-retinal light absorptions.

### Range analysis

The test_retinal_contrast.py program has input values between 0 and maximum luminance. For analysis, the program writes the analytical file <scene_luminance_log _mapped> (8-bit), which records the log-luminance values scaled to <parameter.range>. In other words, by selecting the input range, and logarithmic scaling, calibrated <scene-luminance> and < retinal_contrast> data becomes displayable on a monitor for spatial evaluation.

The calculation and output of the convolution, <retinal_contrast> array, is linear, 64-bit values. The content of the input scene, namely, the population and distribution of luminances determines the range in the <retinal_contrast> output file. The greater the population of high-luminance pixels, the higher the mean-and min-values of <retinal_contrast>. However, since each glare donor pixel sends most of its light to nearby receiving pixels. The scene’s local organization (pattern of scene’s content) affects the local range of <retinal_contrast> values. An Illusion’s pixel population and the separations of max-and min-luminance pixels affects the local ranges of <retinal_contrast>.

### Visual inspection of <*retinal_contrast_log* > images

Human vision’s spatial-image processing suppresses the visibility of luminance gradients (McCann et al., 1974; McCann, 2021b). Visual inspections of <retinal_contrast> images make two flawed assumptions. First, it ignores our vision’s spatial suppression of gradients. Second, it ignores the fact that looking at the calculated image adds a second pattern of actual optical veiling glare to the monitor-displayed calculated glare image. Visual inspection is quantitatively inaccurate. Numerical analysis, and pseudocolor renderings are needed to examine retinal contrast:

- GSF transformed all discontinuous sharp edges into steep retinal gradients.
- Many low-slope gradients are below human detection threshold. Visual inspection does not reveal these gradients.
- Pseudocolor maps, with visible quantization steps, converts subtle luminance gradients into discriminable bands of color, allowing readers to visualize bands of equal-luminance regions, that reveal glare’s nonuniform luminance transformations.

**Figure 3:**
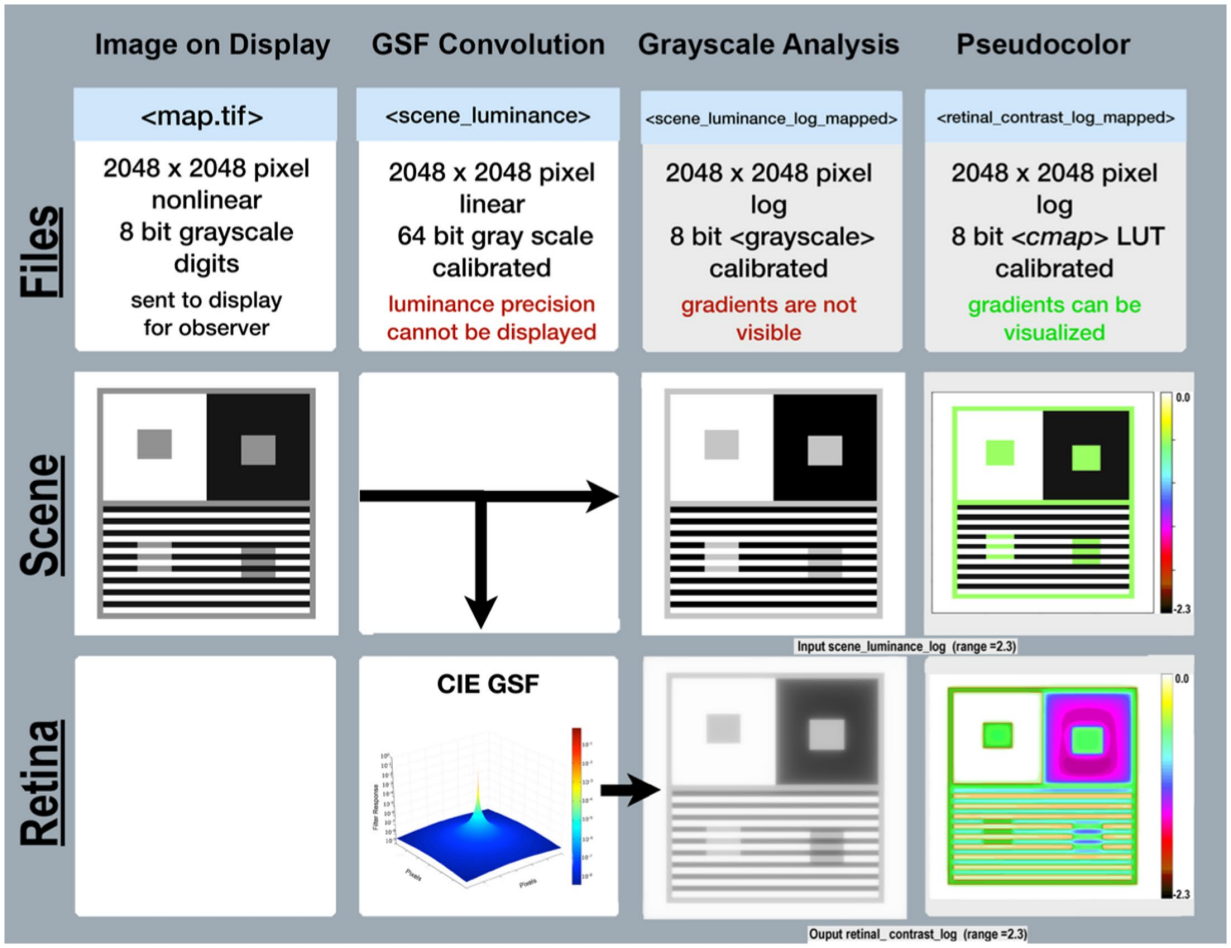
Required data for calculating <*retinal_contrast>*, and analyzing the effects of glare. Columns illustrate the sequential steps in <*test_retinal_contrast.py>*: Image on Display; GSF Convolution; Grayscale and Pseudocolor Analysis. Rows identify the Files; Scenes; and Retina. Files-(top-row) identifies the names, specifications, and precisions at each step. The terms nonlinear, linear, and log refer to plots of cd/m^2^ vs. digit value in the images. The measured luminances from the display were a nonlinear function of Photoshop digits. The program’s calibration step made <*scene_luminance* > linear for the convolution. The analysis of glare used [log_range = 2.3]. Scene-(middle row) illustrates the appearance of the image on the display in the first column; the CIE GSF convolution in the second; the normalized cd/m^2^ input image in the third; and the Pseudocolor visuization of the uniform luminance patches in the fourth column. Note the Color-bar on the right side of this image scene. It plots all 256 pseudocolor samples and identifies the [*log_range*] of the image. Max luminance is White with [*scene_luminance_log* = 0.0] while Min luminance is Black with [*scene_luminance_log* = −2.3]. This Color-bar links the RGB digit values to log luminances. Note that all Gray pixels in Scene(Pseudocolor) have the same Color-bar visualization (green RGB triplet [192, 255, 64]). That triplet is the Pseudocolor output for all grayscale digits in the scene from digit 194 to 197, that calibrates to a log scene luminances range between −0.52 and −0.55. Each Color-band is traceable to log luminance cd/m^2^ values. The second column in Retina-(bottom-row) shows a Pseudocolor 3D plot of convolution kernel for the CIE GSF. The third column shows the grayscale log retinal contrast image used to provide calibrated data for plots, and numerical analysis of <*retinal_contrast* > image segments. The fourth column shows the Pseudocolor image used for visual inspection of the spatial pattern of gradients. Gradients are not visible in grayscale images, but are clearly observed in Pseudocolor. Note Contrast’s large Black surround for the ROI in the third column. Compare it with the Pseudocolor’s visualization of in the fourth column. Peudocolor’s bands of colors reveal the magnitude, and complexity of glare’s gradients.

Figure 3 Files(top-row) describes the specifications of image files used in the program’s sequence (left to right). Scene(middle-row) begins with a reproduction of the Illusion on the display(left column); followed by images used in analysis. Retina(bottom-row) shows images of the pattern of light on the retina scaled to [log_range = 2.3], the input range of the scenes’ luminances.

The CIE GSF uses linear-luminance input data, and high-precision calculation to accumulate all the very small amounts of light from millions of other pixels that fall on each individual pixel. There is no practical method for displaying in this article the actual linear <retinal_contrast> with 4 million pixels at 64-bit precision.

The Pseudocolor renditions allow observers to visualize glare’s gradients of light on receptors. As discussed above, visual inspection does not correlate with quantitative light values. An accurate analysis of the input and output arrays requires numerical inspection and visualization techniques. Readers can identify specific <retinal_contrast_log> values by matching any image pixel’s pseudocolor color to the calibration color map.

### Pseudocolor color-index maps

**Figure 4:**
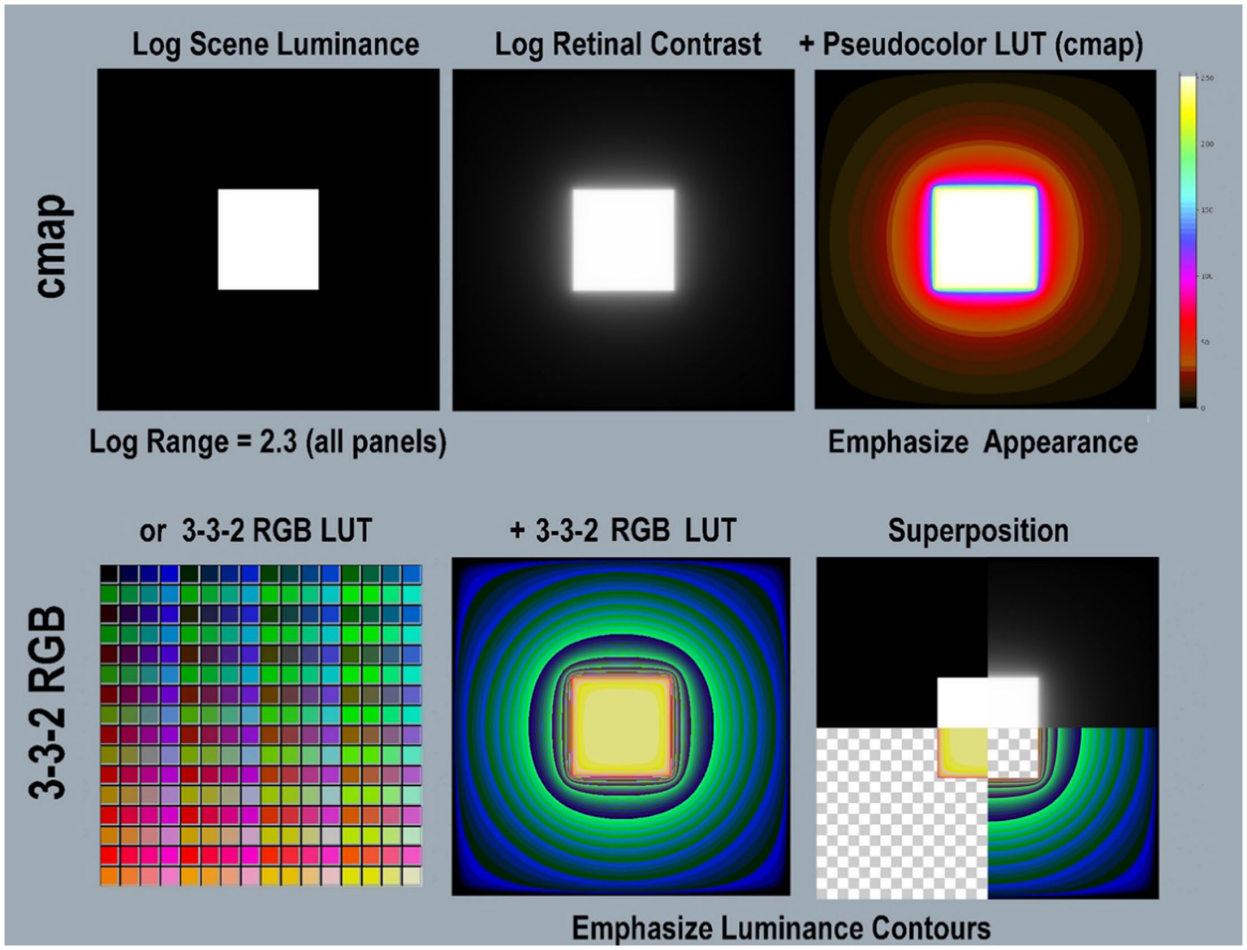
Illustrations of two different Pseudocolor Look Up Tables (LUT). The <c*map.LUT >* (top-row) emphasizes the order of lightness appearances. The left panel shows a 2,049 by 2,049 pixel background (min-luminance) with a centered 601 pixel (max-luminance) square. The left panel is the input file <*scene_luminance_log-mapped* > using <grayscale.LUT>. The middle panel is <*retinal_contrast_log_mapped* > showing the effects of glare. The right applies <*cmap.LUT>,* and shows its color map in its Color-bar on the right. This is used to analyze most of the scenes in this paper. Its color map is encoded in the <*retinal_contrast.py* > program. It used 64 different color bands. (Bottom-row) shows a different LUT, that is implemented in a different way. It has four times more color bands, for better visualization of low-slope gradients. The bottom-left panel shows all 256 different colors in the [*3-3-2 RGB.LUT*] color map, from Min Black [0] to Max Yellow [255]. Its color index emphasizes the visibility of gradients. The bottom-middle panel applies the [*3-3-2 RGB.LUT*] to the retinal contrast file. Note the differences in visualization between [cmap] and [*3-3-2 RGB.LUT*]. The [cmap] rendition preserves the sense of the Lightness separation between Max and Min regions. The [3-3-2 RGB] rendition does not. However, it reveals the presence of gradient throughout the large Min region. Using [3-3-2 RGB LUT] makes it difficult to find the location of the highly visible edge between the Max center and the Min surround. The bottom-right panel identifies the location of that Max/Min input-edge in <[3-3-2 RGB] using the Superposition of four quarter-image sections. The Superposition contains: (1) top-left quadrant is log scene luminance; (2) top-right quadrant is log retinal contrast); (3) bottom-right is background-alone using [3-3-2 RGB]; (4) bottom-left quadrant is square-alone using [3-3-2 RGB], A thin red band locates the Max/Min boundary, that became a gradient after glare.

Figure 4 illustrates two different LUT visualizations using different color-index maps. The Python program includes the pseudocolor [*cmap.LUT*] with 64 color index values, arranged in 8 progressions (top-half). Below it, [*3-3-2RGB.LUT*] is a different kind of color-index map that emphasizes the visibility of gradients. It illustrates glare’s re-distribution in low-luminance regions better than [*cmap.LUT*]. It was applied to retinal contrast using National Institute of Health (NIH) (2021) application ImageJ^®^. It is hard to identify the square’s Max-Min boundary with this LUT. The Superposition panel (bottom-right) identifies the location of that very sharp input-edge using four quarter-image sections. The thin red band falls at max/min boundary that became a steep gradient after glare.

Please take the time to evaluate the spatial patterns caused by glare’s transformations. Please inspect the full-resolution (2MB by 2MB) retinal contrast patterns in Figure 4 file in Data Sheet 1 in Supplementary material.

## Results

**Figure 5:**
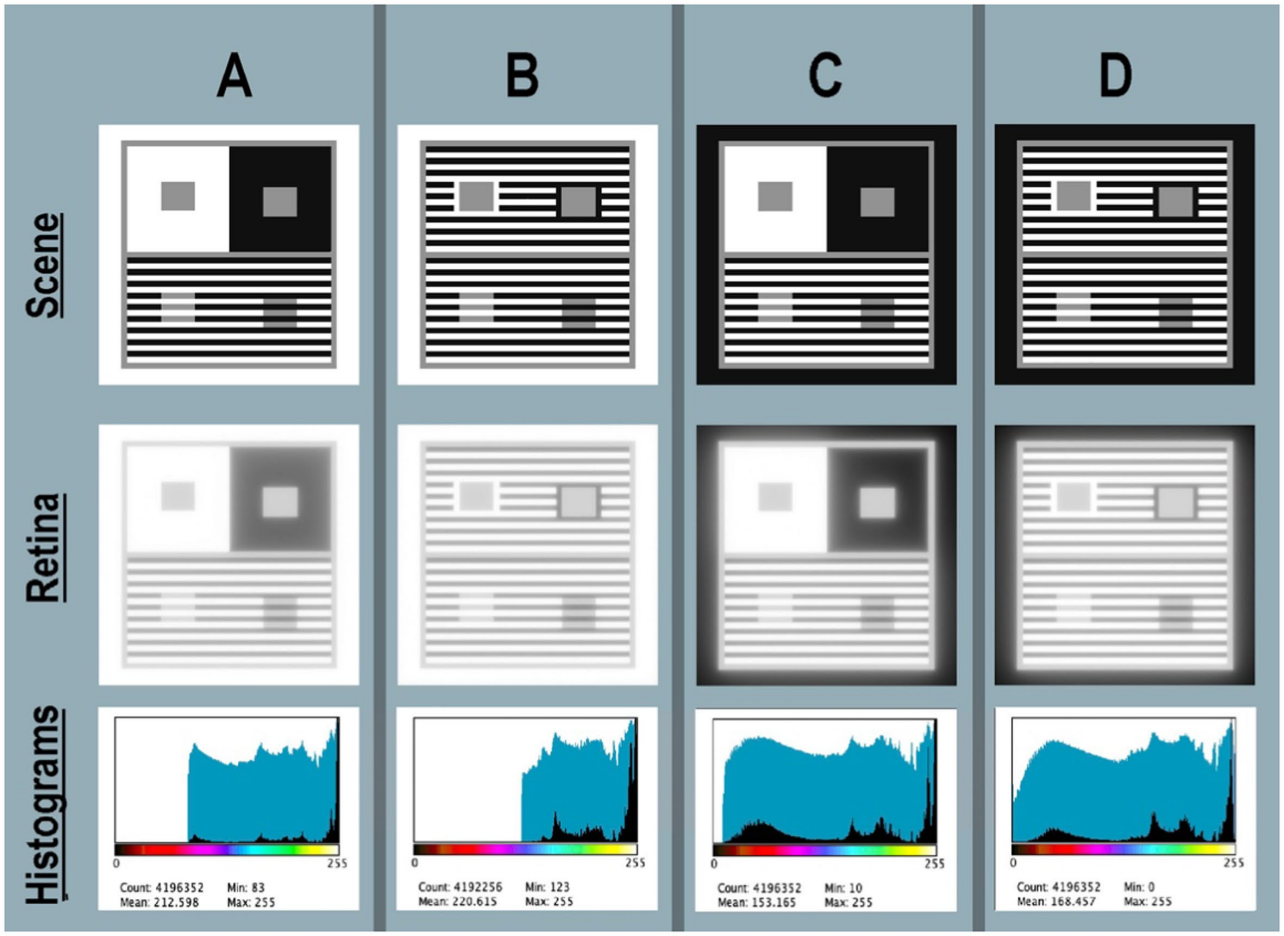
**(A–D)** Four Contrast + Assimilation targets: Scene (top-row) shows four Illusion scenes displayed individually on the computer screen <*map.tif>*; Retina (middle-row) *calculated pattern of* light on receptors <*retinal_contrast_log_grayscale>*; Histograms (bottom-row) linear (black fill) and log (blue fill) histograms of <*retinal_contrast_log_grayscale>*. Above the horizontal axis the color bar illustrates [*cmap.LUT*] pseudocolor mapping. All renditions used parameters [*log_range = 2.3*], [*padding = replicate*].

This article studies glare’s role in three types of Lightness Illusions: Contrast, Assimilation, and Natural Scenes. We begin with four “Contrast + Assimilation” targets in Figures 5A–D. A Contrast Illusion is the top-half, and Assimilation Illusion the bottom-half. In the Scene row, the Contrast, Gray-in-Black surround ROI appears lighter than Gray-in-White. Below Contrast, we add Michael White’s Assimilation Illusion (White, 2010). In Assimilation, Gray-in-Black ROI appears darker.

All Contrast + Assimilation targets are restricted to three scene components: White, Gray, and Black. Identical Gray rectangles (ROI) appear darker in Contrast’s Black surrounds, and lighter in Assimilation’s surround. These different Grays are the result of scene’s spatial content, and spatial arrangements of segments made from uniform Whites and Blacks. The ROI-Grays’ appearances are the consequence of two spatial properties of the scene. First, scene’s histogram, describing populations of all scene pixels (independent of location). Second, size, shape, and location of White and Black segments. In other words, the arrangements of the spatial content in the “rest-of-the-scene” modifies receptors’ responses, and the appearances of GrayROI equal scene_luminances.

Contrast + Assimilation Illusions are robust. Contrast is insensitive to target size (or viewing distance) that changes retinal size (McCann, 1978). Changing viewing distance alters spatial-frequency distribution (intensity vs. cycles/degree). As well, Contrast + Assimilation are insensitive to varying luminance levels. Viewing them in conditions that excite only rods generates the same spatial effects; they just appear dimmer. Viewing color Contrast + Assimilation Illusions in conditions that excite only rods and long-wave cones generates the same color spatial effects, they just appear different hues, and less-sharp than in photopic vision(McCann, 2012, 2021c).

Natural Scenes are much more complex because they do not have any of Contrast’s + Assimilation’s restrictions: uniform scene segments, limited range, uniform illumination. Natural and complex scenes include the interactions of illuminants, reflectances, light emitters, multiple reflections, refractions, shadows, and variable dynamic ranges. The light coming to the eye can be almost any light distribution. Natural Scene Lightness Illusions include experiments that generate different appearances from GrayROI with identical scene luminances.

### Contrast and Michael White’s assimilation targets

First, we made a display’s test target on a display; then, measured its luminances; then, calculated the light on the retina, and finally compared scene luminances with retinal contrasts.

In Figures 5A–D-Scene (top row) show four targets displayed individually on the computer. Each grayscale Contrast + Assimilation scene is a digital array [2,048, 2,048] 8-bit viewed on a Powerbook computer screen at 24 inches, each subtending 10° by 10°. Each pixel subtends 0.24 min of arc. This figure uses a gray-blue background to identify the boundaries of the four targets. A&B targets differ in the size of both Contrast surrounds; A’s is much larger than B’s. This affects the amount, and distribution of glare in Figures 5A,B, but does not change the GrayROI appearances. In Figures 5C,D, outer bands are Black, replacing White in Figures 5A,B. This affects the amount and distribution of glare in both Illusions, but also does not change Illusions’ appearances.

The top row (Figure 5-Scene) shows the images on the display. Placing both Assimilation and Contrast together in each target does not disturb either Illusion. They do not interact. Each does not affect the others’ appearance. Both Contrast and Assimilation appear indifferent to each other. These Illusions add another kind of robustness, and implies that both mechanisms, Contrast and Assimilation, are influenced by relatively local-spatial properties.

#### Numerical analysis of scene input <map.tif>

Scene’s digital values <map.tif > were selected to make the best-looking Illusion on the display. In all four targets the Konica-Minolta CS-100A measurements were: Whites (450 cd/m^2^); Grays (136 cd/m^2^); and Blacks (2.24 cd/m^2^) from a Powerbook Pro XDR display. All targets had a linear range 200:1 [log_range = 2.3]. In all targets, all Gray segments had identical locations, and occupied 14% of each target’s area. In targets A&B, White occupied 57%, and Black 29%. In targets C&D, White occupied 29%, and Black occupied 57% area. These variable patterns of Whites and Blacks caused major changes in glare, shown in retinal_contrast’s histograms. However, these changes in the “rest-of-the-scene” do not alter the appearances of the GrayROIs.

#### Appearance of calculated *retinal_contrasts*

Figure 5-Scene recreates the appearances on the display. The Python code combines the Scene’s design with its luminance calibration to make convolution’s input array (normalized linear luminances) at 64-bit, double precision. The convolution calculates high-precision retinal_contrast values. Three additional steps are needed to analyze the output: precision (64 to 8-bit) for display: mapping to input’s range; and logarithmic scaling. Figure 5-Retina(middle-row) shows [log10_range = 2.3] output. Retina’s logarithmic data optimizes grayscale and pseudocolor visualizations. The <retinal_contrasts_log_grayscale> images have apparently less-sharp edges, and have less range of light. Glare has rounded the scenes’ square-wave edges that appear sharp when viewing them on the display (Figure 5-Scene).

Vision’s spatial-image processing has synthesized these sharp-edge appearances from the retinal image. Thinking about the observer’s appearances of Retina’s fuzzy images, recalls many relevant facts. For example, cones in the fovea have approximately 1 min of arc spacing. However, stereo depth can resolve 2 seconds of arc in retinal disparities. Observers with good binocular vision can have stereo-acuity thresholds as low as 2 s of arc, and 80% have 30 arcsec thresholds (Howard and Rogers, 2002). In hyper-acuity, optimal discrimination threshold for relative positions of two features in the fovea is a few seconds of arc(Westheimer and McKee, 1977). Vision’s spatial-image processing is more precise than cone spacing. Hubel and Wiesel (1965) discovered that Visual Cortex neurons respond to edges, while they are unresponsive to spots of light. Zeki’s v4 cortical color cells respond to complex images, but not to “spots of light” (Zeki, 1993). Vision uses spatial-image processing to synthesize the appearance of sharp edges. Today’s powerful AI object recognition techniques use Hubel & Wiesel, and edge-detection techniques in early stages. Edges lead to shapes, that lead to identifying objects. Engineering development of “Event Cameras,” that mimic human image processing are wide spread(Curtis, 2022). These observations, as well as innumerable others since the 1960s, changed vision research and electronic imaging by mimicking human spatial processes in Retinex, Object Recognition and Neuromorphic Cameras. Vision, human and virtual, went from using scene-independent models of pixels to scene-dependent models of images.

#### Numerical analysis of calculated *retinal_contrast*

Figures 5A–D—Histograms plots linear and log histograms of Retina. All histogram plots are [log_range = 2.3], equal to input range. Recall that the scene luminance input images have histograms (not shown) of only three spikes at digits 255, 145, and 21. Glare has re-distributed those spikes into four very different light patterns. Target A is the most familiar version, viewing the Illusion on a white paper, or white screen. Glare reduces RetinaA to [67% log_range]. The outer white band adds enough glare light to the large Contrast Black surround to set the abrupt lower range limit at digit = 83. Target RetinaB replaces Contrast’s large Black, and large White surrounds with Assimilation’s stripes. Here, Contrast’s Gray test areas are still surrounded by Black, and by White segments, but they are alternating bands. These changes greatly reduced the average angular distances between Whites (glare net donors) and Blacks (glare net receivers). The result of closer glare sources decreased RetinaB to [52%log_range]; half that of the input scenes.

In Targets SceneC and SceneD the outer band is Black. The program’s [padding = replicate] setting for outermost pixels calculates displays in a darkroom on a Black background. Replacing White with Black outer edge, and decreasing the size of Contrast’s surrounds in D caused a major increase in range of retinal_contrast_log. The abrupt lower limit of the minimal retinal_contrasts in RetinaA and RetinaB resulted from nearby White segments in the outer edge and Contrast regions. Here, in RetinaC and RetinaD retinal ranges increase because there is less glare light in Blacks. Target RetinaC range is [95%log_range]; Target D range is [100%log-range]. Overall, these four targets varied from 52% in RetinaB to 100% in RetinaD.

Numerical analysis of calculated retinal contrast describes two distinctly different types of targets: one with a max-luminance outer band (RetinaA, RetinaB); the other with a min-luminance band (RetinaC, RetinaD). Nevertheless, observed appearances of Contrast and Assimilation are constant, despite major changes in retinal contrasts’ patterns, and the subsequent responses of retinal receptors.

Numerical analysis of retinal contrast in Figures 5A–D Retina shows that all four Contrast Illusions exhibit Glare’s Paradox; namely, regions-of-interest Gray-in-White appear darker despite larger amounts of glare light. And Gray-in-Black ROIs appear lighter despite less glare light.

For example: in top-half Contrast(A) GrayROI rectangles have uniform <scene_luminances>. After glare those rectangles become ranges: Gray-in-Black[68%–83% log-range] retinal_contrasts, and [81%–93% log-range] in Gray-in-White. The large white surround adds more glare light to its GrayROI. The psychophysical challenge is to understand why more-light in GrayROI-in-White in all Figures 5A–D—Retina look darker in Scene.

Assimilation does not exhibit Glare’s Paradox; more-light in GrayROI-in-White in all Retina(A,B,C,D) look lighter in Scene(A,B,C,D).

Glare created four different log range outputs. To understand different spatial patterns of light re-distributions, we use pseudocolor LUTs to visualize the gradients of light on receptors.

Please inspect the full-resolution <grayscale> retinal contrast patterns in Figure 5 file in Data Sheet 2 in Supplementary material. Gradients are nearly invisible.

#### Pseudocolor analysis of calculated *retinal contrast*

**Figure 6:**
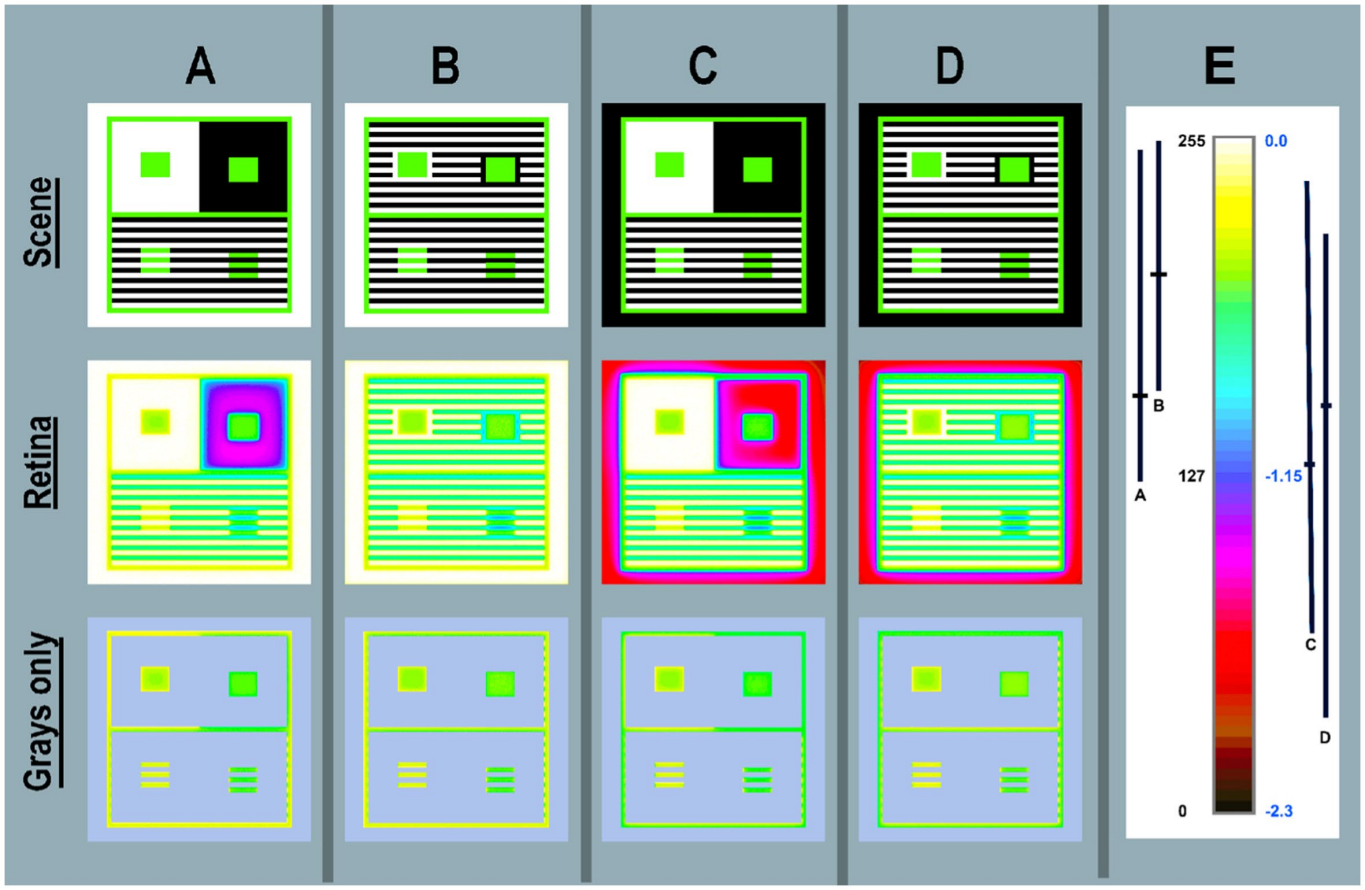
Pseudocolor renditions of the four Illusions shown in <grayscale> in Figures 5 (A-D). Column (**E**) shows [cmap.LUT] color index map. Scene (top-row) < *scene_luminance_log_cmap >* images [*log_range = 2.3*]. Retina (middle-row) calculated <*retinal_contrast _log _cmap >* images. Grays only (bottom-row) copies of Retina are covered by a light-blue mask over all the max-and min-luminances. This leaves Grays only pixels in all four Illusions. Enlarging the Grays Only image illustrated glare’s distortions of uniformity in GrayROIs. Column **(E)** adds an enlarged color-bar showing the Pseudocolor conversion from digits to color patches. The range of digits is [0, 255]; the range of *log_retinal_contrast* is [−2.3, 0]. The black vertical lines **(A–D)** plot the ranges of <*log _retinal_contrast >* of all Black pixels (*scene_luminance* = 2.2 cd/m^2^) in the each Illusion. The horizontal line in each range is its mean *log _retinal_contrast* value. Every Black glare-receiving pixel value varies with the angular distances between itself and all the donating White and Gray pixels. The changes in spatial position of these scene elements causes the dramatic variability of Black retinal contrast values. Nevertheless, they have identical rich black appearances on the display (Figures 5A–D-Scene).

Figure 6 maps images in Figure 5 using pseudocolor. All four targets have only three luminance values: (max-White, Gray, min-Black) illustrated by images in Figures 6A–D-Scene. Pseudocolor renders max = white; gray = green; min = black. Figure 6-Retina applies the same LUT to retinal images. As expected, glare has minimal, but apparent changes in Whites’ pseudocolor segments. Many Whites that are adjacent to Black become yellow at the edge.

The substantial, but subtle effect on Gray scene segments is seen best by studying the Grays only row. The constant Gray borders in all Scenes around Contrast and Assimilation Illusions shows that retinal_contrast has a different border patterns in A,B,C,D. Contrast’s GrayROI rectangles are affected by the traditional large White and Black surrounds. The outer White and Black bands and the replicate option adds to scene-dependent variability.

The most striking result from these four targets is the retinal_contrast maps of Black regions. These constant, uniform scene segments became highly variable, nonuniform, scene-specific retinal contrast values. The ranges of Retina Black are plotted in Figure 6E beside the color bar. The effect of glare on Blacks is very large and highly variable. The appearances of all Black segments are constant, but the amounts of light on receptors are variable: (A)log_range[49%–98%]; (B)log_range[62%–99%]; (C)log_range[26%–93%]; (D)log_range[15%–86%].

Scene has [log_range = 2.3]; and Retina(Blacks-Only) has [log_range(A) = 1.1]; [log_range(B) = 0.9] [log_range(C) = 1.5] [log_range(D) = 1.7]. Scenes(A,B,C,D) are not million-to-one range HDR targets; they are normal range 200:1 displays. How does vision generate nearly identical appearances from such variable information in receptor responses? What mechanisms can calculate these results?

By addressing the actual image on the retina, we can no longer assume a zero-glare hypothesis in “normal” scenes. That zero-glare hypothesis made us believe that designs of Illusions were appropriate stand-ins for uniform-surface objects in the world that had recognizable independent shapes and interpretable perceptual properties. Real retinal images require mechanisms that finds these shapes in each illusion’s nonuniform unique retinal gradients. Then, these mechanisms must find a way to make them appear identical.

Glare does not alter the fundamental proposition of Illusions, namely that equal scene_luminances do not generate equal appearances. However, glare creates a unique spatial pattern for each of the four Contrast + Assimilation targets in (Figure 6). Observers do not see glare’s re-distribution of light. Nevertheless, glare is scene specific. There are no accurate short-cuts modeling these targets because the GSF never reaches an asymptote. Short-cuts based on highly simplifying assumptions can be misleading. Models of glare must incorporate all the individual scene-dependent contributions from all the other pixels.

In summary, Figure 6 visualizes the retinal light pattern that becomes the array of receptor responses. That pattern shows the scene-dependent transformations of scene_luminances. Distortions of GrayROI luminances, make them unequal retinal_contrasts. This affects the asserted logic of a Lightness Illusion, that GrayROIs are equal stimuli. The range distortions for GrayROIs are small. However, that range is very large for Blacks, even though the Scene’s range is limited to 200:1.

The summary from section Contrast and Michael White’s Assimilation Targets is very simple. Figure 5-Scene shows all four Contrast + Assimilation Illusions on the display. They are made of only 450, 30, and 2.2 cd/m^2^ regions. Figure 6-Scene shows the spatial distribution of scene_luminances. Figure 6-Retina shows glare’s redistributed light patterns on receptors.

Please inspect the full-resolution pseudocolor retinal contrast patterns in Figure 6 file in Data Sheet 3 in Supplementary material. Gradients are clearly visible.

### Contrast and Todorovic’s assimilation targets

**Figure 7:**
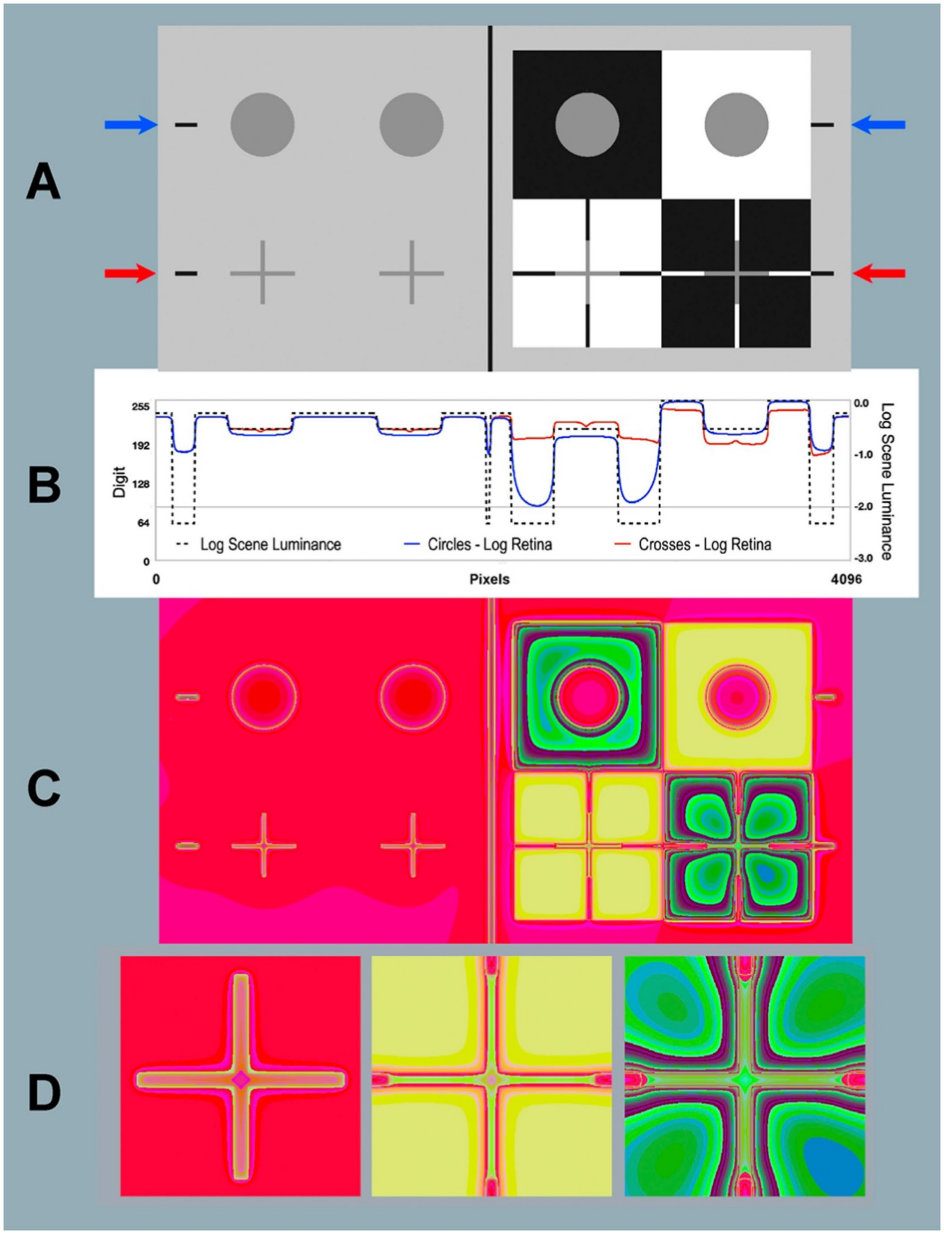
Contrast and Todorovic Assimilation targets. **(A)** Scene: Image [*log_range = 2.3*] displayed on computer screen (top-half is Contrast; bottom-half is Assimilation). **(B)** Horizontal log luminance plots through the centers of the circles and crosses. Horizontal log scene luminances plots are identical in top Contrast and bottom Assimilation (dashed black line). Log retinal contrasts are different: circles (blue line at blue arrows); crosses (red line at red arrows). **(C)** Retina: Calculated log retinal contrast using [*padding = replicate*] and Pseudocolor [3–3-2RGB LUT], [*log_range = 2.3*]. **(D)** Enlargements of Retina Assimilation crosses: Gray-in-Gray surround (left); Gray-in-White surround (middle); Gray-in-Black surround (right). The 3-3-2 RGB LUT reveals equal luminance regions in Retina. Recall that the Scene is made up of only 4 uniform luminance (White, Gray cycles and crosses, Black, and background). Glare transforms Scene uniformities in very complex nonuniform patterns on the Retina. Blacks show the largest glare distortions. These luminance distortion patterns are invisible when viewing the display in **(A)**.

In Figure 7A we have eight identical gray luminances (four circles-top and four crosses-bottom). On the left side these grays (uniform background) all appear the same lightness. On the right, the four grays (different backgrounds) have different appearances.

On top-right we see the background pattern has the traditional Contrast Illusion surrounds: Black (lighter appearance); White (darker appearance). Below that, Todorovic (1997) Assimilation is scaled to fit Contrast. In Assimilation apparent-Gray circles are behind slits in White, and Black foregrounds. In this spatial arrangement, the mostly-White ground makes Gray appear lighter, mostly-Black makes Gray appear darker.

We used Python code to calculate the <retinal_contrast> of Figure 7A 4,096 × 2,048 pixels; 8-bit display. The viewing_distance was 24 inches, subtending 20° by 10°. Each pixel subtends 0.24 min of arc.

#### Numerical analysis of *scene luminance* and calculated *retinal contrast*

Glare changes the output range of linear retinal contrast to 62:1, compared with the input range of 200:1. The blue arrows and red arrows in Figure 7A indicate the locations of two horizontal digital (1 pixel high) scans across the input and output images of the Contrast Illusion’s Gray circles and Assimilation crosses.

The dashed-black line (Figure 7B) plots the input scene luminance values. These inputs are identical at both blue and red arrows. They plot input, and illustrate edge sharpness in displayed scene_luminance. They pass through a portion of all four types of scene segments (W, B, G, and background).

Along the blue scan, glare has reduced retinal_contrast to [log_range = 1.7]; and along red scan Assimilation [log_range = 0.75]. Linear values are[Scene range = 200:1; Contrast range = 50:1; Assimilation range = 5.6:1]. Assimilation segments have lower range and more rounded retinal edges.

In Figure 7B blue-line plots retinal_contrast_log through the middle-line of all gray circles. The red-line plots crosses’ middle-line of horizontal arms. The red and blue scans of GrayROIs are different. In uniform light-gray background, Grays-in-background crosses (red) have slightly more scattered light than circles (blue). On the right-side (Illusions), Assimilation’s White foreground adds the most glare light. Contrast’s circle in Black surround received the least amount of glare in all scene segments. Its large Black surround becomes a large asymmetric U-shaped gradient.

In Figure 7A both Circles are examples of Glare’s Paradox. The GrayROI-in-White appears darker with more glare than GrayROI-in-Black; that appears lighter. Todorovic’s Assimilation has a very different glare pattern. Here, Todorovic’s Cross-in-White foreground is maximal glare and Cross-in-Black is minimal. These glare-induced changes are much larger than Contrast, with opposite effects. Assimilation’s glare adds more glare to apparently lighter segments; and less to darker ones. Again, Assimilation does not exhibit “Glare’s Paradox.”

#### Histograms of gray-ROI’s in contrast and Todorovic assimilation targets

**Figure 8:**
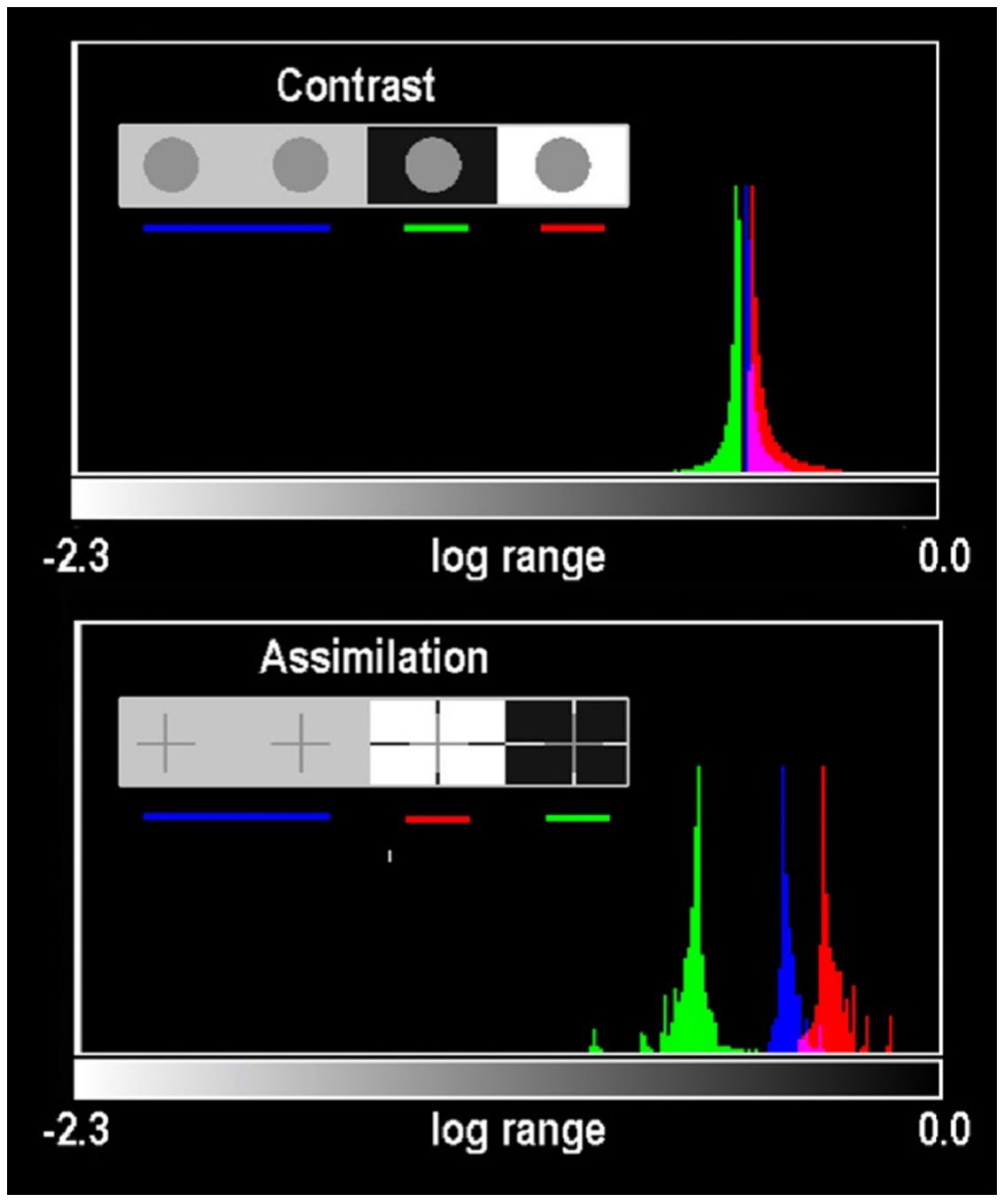
Histograms of all Gray pixels in Contrast (circles) and Todovoric Assimilation (crosses) in different backgrounds. Plots of *retinal_contrast_log* scaled to l*og_range =* [−2.3,0.0] vs. pixel count. The vertical axis is a linear count (256 bins). Each histogram is normalized to its own peak. Gray-in-Black surrounds are green; Gray-in-gray are blue; Gray-in-White are red. In Assimilation crosses, glare adds more light to Gray segments that appear lighter in White, and the least light to Grays that appear darker in Blacks (Figure 7A). The opposite happens in the Contrast’s circles, showing Glare’s Paradox.

Figure 8 plots histograms of all Gray pixels in circles and crosses in different backgrounds. Contrast and Assimilation differ in ranges and distributions of glare. In circles (Figure 7A; top) the max/min edges are 46 min radius from their centers. The crosses are 10 times closer to max/min edges (4.2 min at nearest pixel). In Assimilation, glare adds the most glare to Gray-in-White pixels(red-plot). Grays-in-Black(green-plot) have the least glare. In Assimilation, glare adds more glare to Grays that appear lighter, and the least to those that look darker. The opposite happens in Contrast’s circles, showing Glare’s Paradox.

#### Pseudocolor analysis

Contrast and Todorovic Assimilation have uniform scene_luminances with perfect square-wave edges. There are no gradients in this input digital image. In retinal_contrast all sharp edges become a wide variety of different slope gradients. Figure 7C is a pseudocolor rendition of <retinal_contrast_3-3-2 RGB.LUT>. Glare transforms uniform scene Blacks into an assortment of gradients on the retina. Figure 7C shows dramatic local-spatial-transformations of the “equal scene Grays.” The [3-3-2 RGB.LUT] was designed to visualize numerically uniform scene segments. It does not preserve apparent lightness, as [cmap.LUT] does. Four uniform scene_luminances, become this very complex pattern of receptor responses.

Todorovic crosses are made of lines that are 380 pixels long, and only 25 pixels wide. When viewed at 24 inches these lines subtend 1.5° by 6 min of arc. Figure 7D shows enlarged glare gradients surrounding crosses. The sharp pseudocolor edges in Figure 7D allow us to visualize gradients that are invisible to us in grayscale images. The resolution of these computations was chosen to be slightly higher than foveal cone-mosaic spacing, but lower than spatial-processing performance in Hyperacuity and Stereo Acuity. This image describes patterns of light on receptors. There are many subsequent variables that follow in the visual pathway to appearance: observer acuity, rod and cone sampling, receptive-field organization, cortical-multi-resolution fields (image domain), or spatial-frequency channels (Fourier domain), and neural-spatial processing. These steps are beyond the scope of this article.

Intraocular glare upsets Lightness Illusions “null experiment.” Glare redistributes scene’s light patterns. These retinal patterns are unique in every scene because they respond to the entire pixel population (histogram), and each pixel’s relative positions to each of the other 8-million pixels. The complex-spatial patterns made with Pseudocolor LUTS suggests how difficult it is to analyze appearances if we restrict ourselves to using single-pixel analysis of data. Every pixel’s correlation with scene luminance is altered before light reaches retinal receptors. Predicting appearances based on scene-independent models (extensions of silver-halide films and Colorimetry principles) is an extraordinary challenge. The light falling on a single pixel (quanta catch, or CIEXYZ) is an unreliable prediction of its appearance. The only condition in which single-pixel data correlates with appearance is the special case of perfectly uniform segments, in uniform illumination, in uniform constant “rest-of-the-scene” (McCann, 2017, 2020). We need to recall that appearances are the result of spatial comparisons. Post-receptor neurons in the visual pathway perform these spatial image processing steps. Illusions make the point that appearances are the consequence of spatial comparisons involving “the-rest-of-the-scene.”

Please inspect the full-resolution <grayscale> and pseudocolor retinal contrast patterns in Figure 7 file in Data Sheet 4 in Supplementary material.

### Edwin Land’s black and white Mondrian

**Figure 9:**
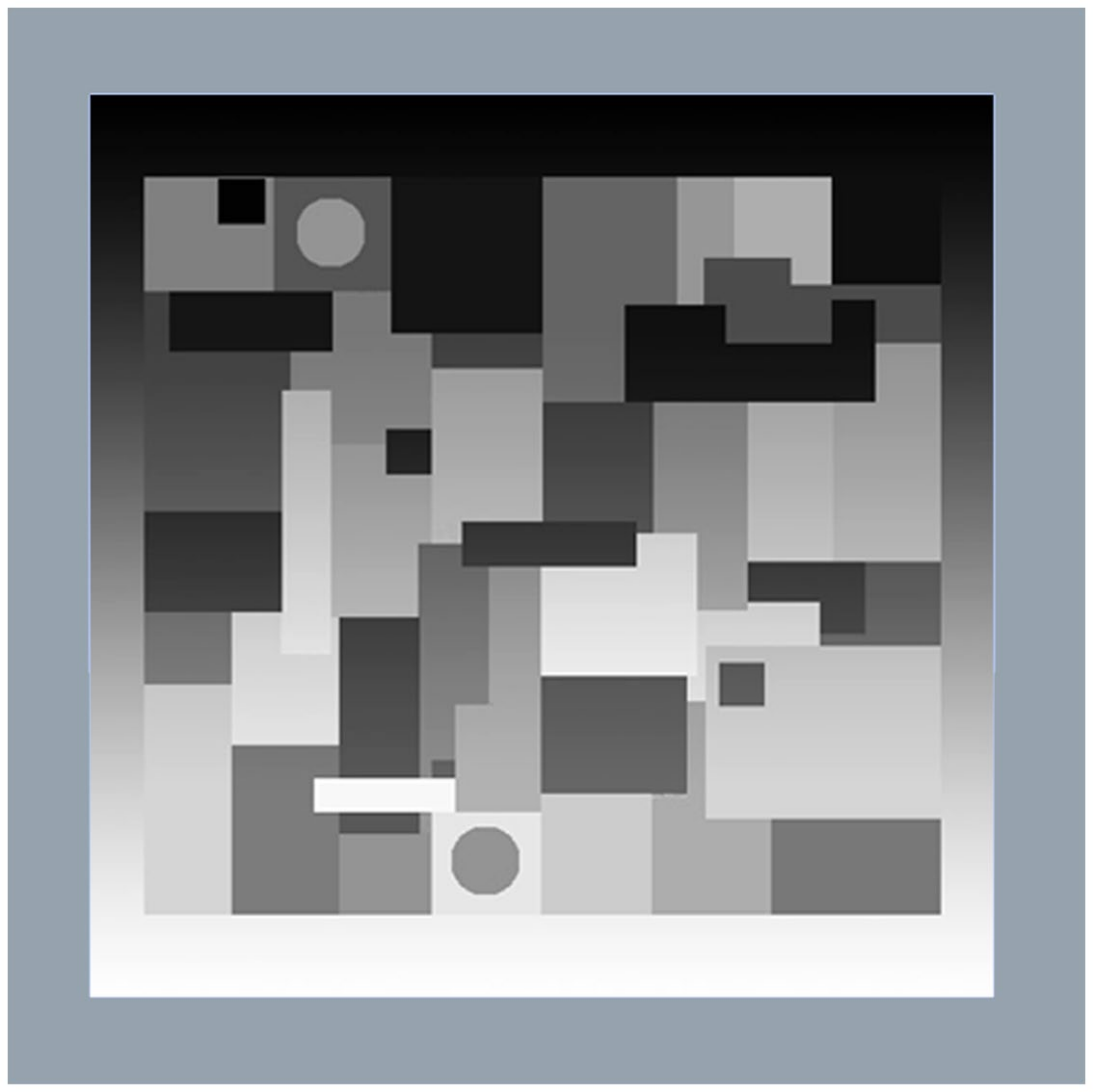
Illustration of Land’s B&W Mondrian. Edwin Land’s demonstration of his Black and White Mondrian (Ives Medal Address to the Optical Society of America in 1967).

Figure 9 is a simulation of Edwin Land’s constructed Natural Scene. The original experiment used over 100 achromatic-matte-surface papers, intentionally made with different paper sizes and shapes to avoid afterimages (Daw, 1962; Land and McCann, 1971). It used an illumination gradient (bright-at-bottom), (dim-at-top). Land selected two paper ROIs (circles in this simulation): high-reflectance paper at the top, and low-reflectance at the bottom. He adjusted the gradient of light so luminances from these papers had identical scene luminance circles. The top circle appears near white; bottom is much darker. Land demonstrated that both White and Black appearances were generated by the same light, at the same time, in the same scene. In 1967, this observation, made by the OSA audience, was unique. Land’s actual demonstration had greater range of light, and greater range of appearances than Figure 9. In Land’s HDR scene construction, paper at the top appeared whiter; and bottom paper appeared blacker.

**Figure 10:**
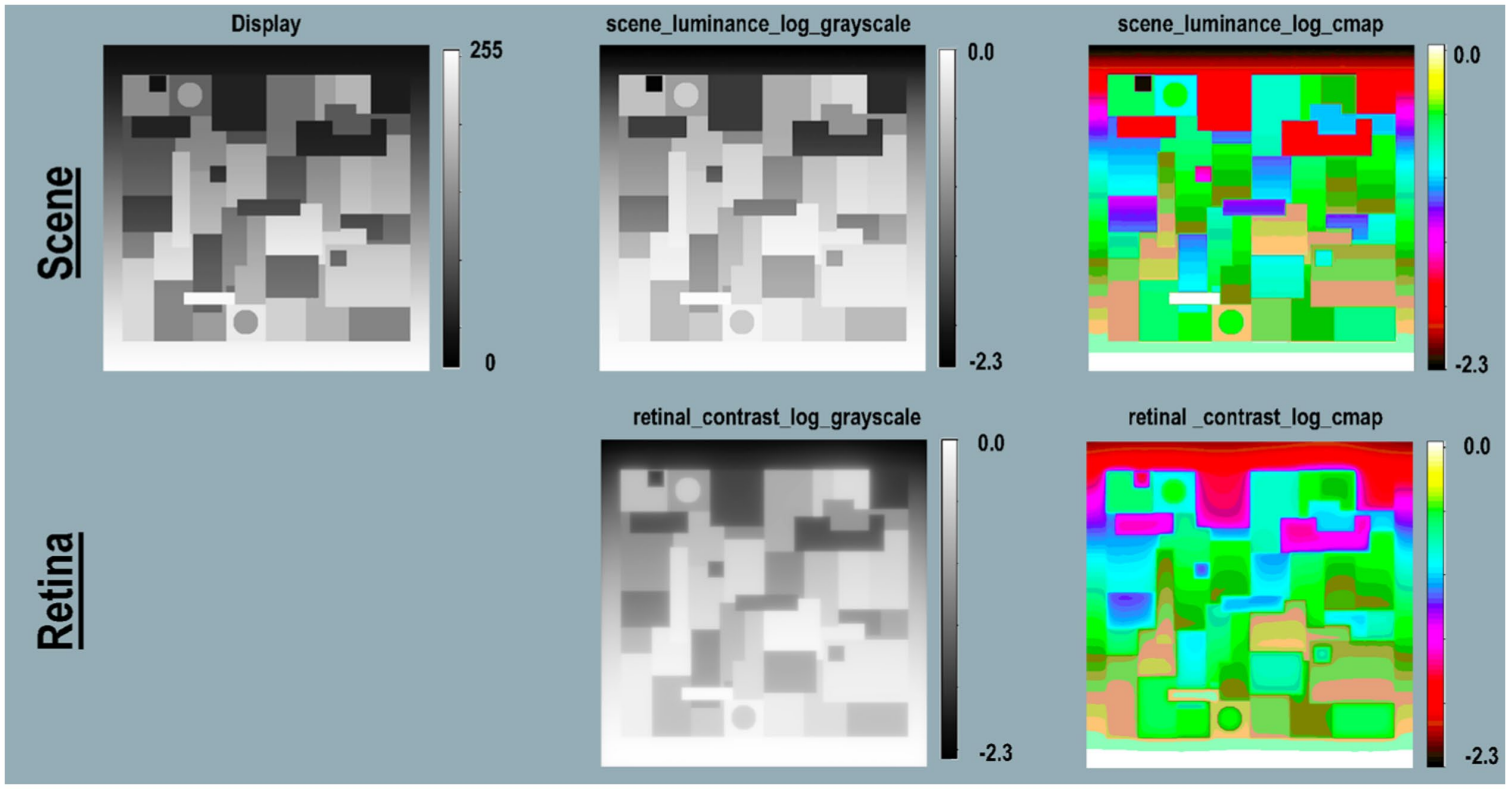
Land’s B&W Mondrian. Scene (top-row) Mondrian on display; *scene_luminance _log_grayscale, and scene_luminance_log_cmap.* Retina (bottom-row) *retinal_contrast* using *same LUTs.* All Figure 11 calculations used parameters [*log_range = 2.3*], [*padding = replicate*].

Figure 10-Scene (top-left) shows the Mondrian on the display; log grayscale, and pseudocolor renditions. Below are the *retinal_contrast_log _mapped* images. Pseudocolor shows clearly how luminance was affected by the gradient of illumination. The scene’s gradient is barely detectable in the grayscale image. The retinal contrast data show small amounts of spatial distortion by glare at the Mondrian’s top. Each circle center has *scene_luminance* equal to [80% log_range]. After glare, the *retinal_contrast* top-circle (appears lighter) is [78% log_range]. The lower darker circle is [84% log_range]. Glare increased *retinal_contrast* of the darker circle. This is another example of Glare’s Paradox. Neural spatial processing overcomes the effects of glare by making the circle with increased receptor responses appear darker.

Please inspect the full resolution B&W Mondrian retinal contrast patterns in Figure 10 file in Data Sheet 5 in Supplementary material.

**Figure 11:**
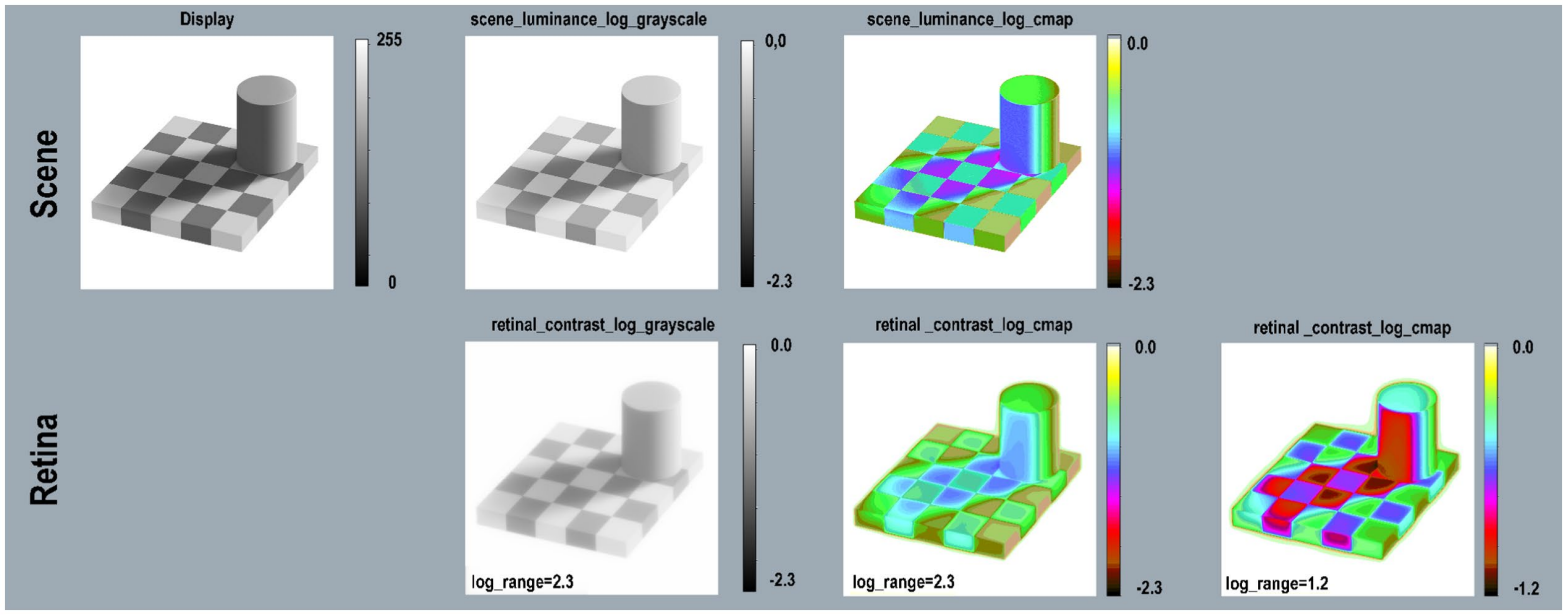
Checkershadow Illusion—Scene (top-row) reproduces the image on the display; *scene_luminance_log_grayscale;* and *log_cmap.* Retina (bottom-row) *retinal_contrast* using the same mapping. All calculations used parameters: pseudocolor [*cmap.LUT*], [*padding = replicate*]. The first three columns used [*log_range = 2.3*]. The extended White surround for the Tower and Checkerboard raised the mean *retinal contrast* values and reduced the total [*log_range* = 1.2]. The final column on the right used [*log_range = 1.2*] to get a better rendition of *retinal_contrast* values in this illusion.

### Adelson’s Checkershadow illusions

Ted Adelson (1995) made a synthetic target called the Checkershadow® Illusion. Land never called his Black and White Mondrian experiment an Illusion. The B and W Mondrian, and the Checkershadow are, in fact, the same experiment. They are made of highly visible edges, and hard-to-see gradients. Land used luminance and appearances measurements in the B&W Mondrian experiment to propose a bottom-up model of calculating apparent Lightness sensations. As Land pointed out, Lightness does not always correlate with reflectances (Land, 1974). In this research, Lightness is defined as appearance measured by observer matches to a standard complex target (McCann et al., 1970). The work developed into a multi-resolution application, and hardware implementations (Frankle and McCann, 1983; McCann, 1999, 2004) that calculated Lightness appearances that correlated with observer matches (McCann and Rizzi, 2011, pp.293–337).

Land believed that accurate illumination was “unknowable,” as he wrote in the last sentence of his Ives Medal Address (Land and McCann, 1971). Given the array of all scene luminances, Retinex’s approach was to build appearance by emphasizing edges and minimizing gradients. These Land and McCann, and other Retinex algorithms modified the statistical properties of scene luminance arrays (McCann and Rizzi, 2011).

Adelson’s (1995) version of edges and gradients (Checkershadow®) is in-practice the same as Land’s B&W Mondrian. Adelson introduced digital gradients attributed to illuminance, and digital edges attributed to reflectance. Adelson used a different definition of Lightness, namely “Lightness is defined as the perceived reflectance of a surface. It represents the visual system’s attempt to extract reflectance based on the luminances in the scene.” Adelson claimed that “… illuminance and reflectance images are not arbitrary functions. They are constrained by statistical properties of the world.” (Adelson, 2000). Land and McCann defined Lightness as observer appearance matches to a standard complex scene (McCann et al., 1970; Land and McCann, 1971; Land, 1974). Later, Adelson’s defined Lightness as a surface perception(Adelson, 2000).

Since this article has limited scope, it cannot resolve which set of statistical properties are the better framework for appearance: bottom-up statistics of each scene, or top-down statistics of the world. The article will continue with the study of effects of glare on Adelson’s Checkershadow’s retinal_contrast (Figure 11).

The Checkershadow has edges connected by gradients. The biggest difference between Mondrian and Checkershadow experiments is the large-White surround, resembling a beach scene (McCann, 2014). The Checkershadow has mean scene_luminance of 50%log_range compared with 30% for B&W Mondrian.

That White surround reduces Checkershadow’s scene_luminance [log_range = 1.6] to retinal_contrast [log_range = 1.2]. Adelson’s specified square (Checkershadow, top-edge) ROI appears darker. Its retinal_contrast values vary from [72% to 90%log_range]. The lighter-central square varies from [65% to 71%log_range]. The “Illusion” overcompensates glare because receptor responses to “darker square” are greater than those of “lighter square.” It is another example of Glare’s Paradox.

Please inspect the full-resolution Checkershadow retinal contrast patterns in Figure 11 file in Data Sheet 6 in Supplementary material.

#### Glare’s paradox

**Figure 12:**
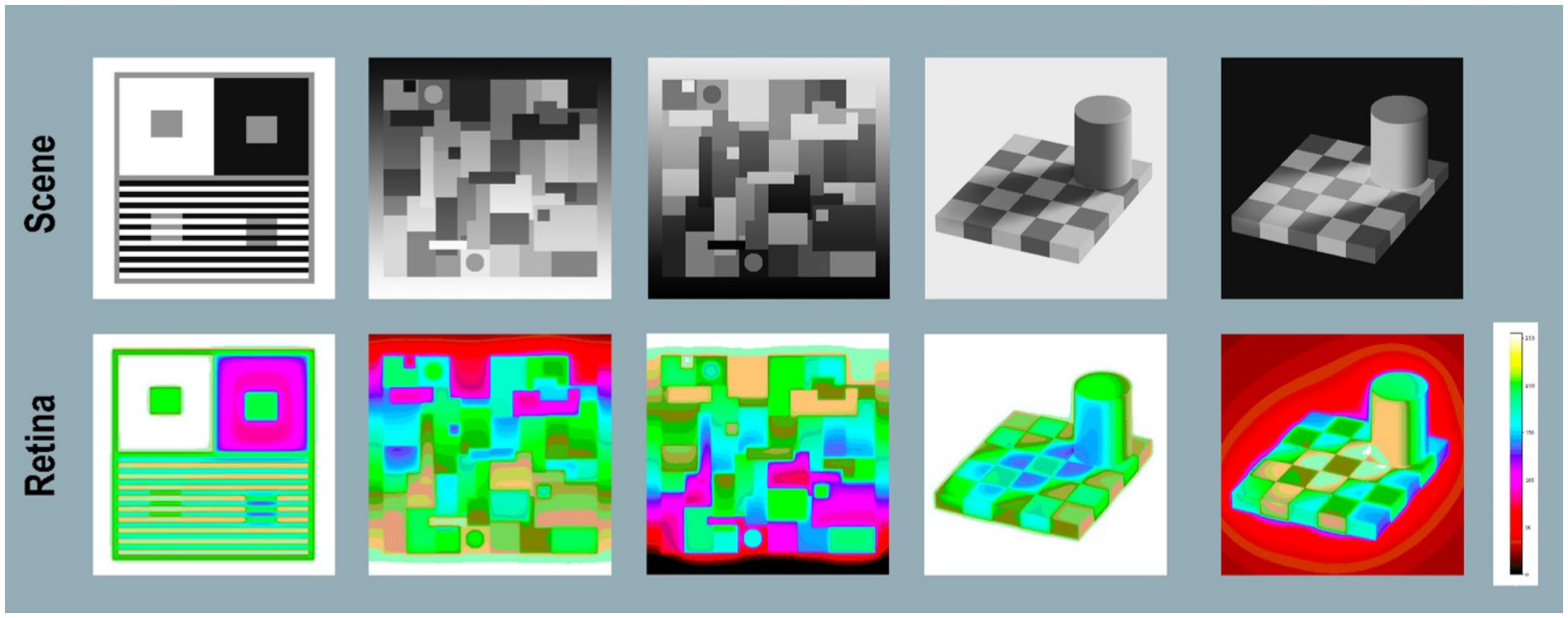
Glare’s Paradox-Scene: (top-row) shows Appearances of: Contrast, Mondrian [positive and negative], Checkershadow [positive and negative]. Retina:(bottom-row) pseudocolor rendering using [*cmap.LUT*]. On the far right is a plot retinal contrast digit value [0,255] vs. pseudocolor samples used to identify *retinal _contrast_ log* values. In total, this article calculates the *retinal_contrast* image for 9 Lightness illusion scenes. All 9 scenes contained GrayROI segments that showed Glare’s Paradox. In the 5 scenes that contained Assimilation Illusions, none of their pairs of GrayROI showed Glare’s Paradox.

Figure 12 (top) shows the appearance of the Contrast, B&W Mondrian, Checkershadow computer displays. It adds Negative displays of B&WMondrian and Checkershadow made with (Photoshop’s® Invert function). Negative Illusions work very well. The Mondrian has a different pattern with top-illumination. The “shadow” in Checkershadow now appears to emit light. The [cmap.LUT] (Figure 12, bottom-row) displays the complexity and variable range of Glare Paradoxes.

In the Negative Mondrian, the top-darker circle has retinal_contrasts varying from [70%–79%*log_range*]. The bottom-darker circle varies from [65%–71%*log_range*]. In the Negative Checkershadow, the central-darker ROI has retinal contrasts varying from [86%–92%*log_range*]. The top-lighter square varies from [78%–85%*log_range*]. Appearances of both GrayROIs in Negative Illusions (Mondrian and Checkershadow) overcompensate glare.

Five Contrast Illusion targets, Positive-and Negative B&W Mondrians, and Checkershadows are all examples of Glare’s Paradox. Namely, darker GrayROIs appearances have more glare light. These darker ROIs are in local regions with higher-than-average scene_luminances. The sequence of observations is [greater average scene_luminance region ➜ greater glare ➜ smaller edge ratios ➜ higher-slope visual response function ➜ darker appearance].

Studies of glare in HDR scenes (McCann and Rizzi, 2011) showed extraordinary reductions of retinal-dynamic range in maximal-glare scenes. The input scene has [*log_ range* = 5.4]; after glare [*log_range* = 1.5] (McCann and Vonikakis, 2018). Vision’s net response function to light on receptors varies with scene content. Vision has limited-range (high-slope) visual-response function in high-glare scenes. These darker Glare Paradox regions in Lightness Illusions, affected by glare, produced lower-range retinal_contrast, and have appearances associated with high-slope visual-response functions.

Glare’s Paradox exhibits reciprocal properties for GrayROIs that appear lighter. In all Contrast and Natural Scene examples: the sequence of observations [lower average scene_luminance regions ➜ less glare ➜ larger edge ratios ➜ lower-slope visual response function ➜ lighter appearance].

Glare’s Paradox is not found in Assimilation segments. Glare adds more glare light to segments that appear lighter; less light to segments that appear darker. The angular separation between max and min are smaller, and local retinal_contrast range is smaller. Glare assists Assimilation’s change in appearance. Assimilation Illusions improve with smaller angular size, unlike Contrast Illusions where observer matches are constant with changes in size. (McCann, 1978).

Region-dependent visual response functions could account for neural-spatial image processing that tends to cancel glare. Examples of region-dependent image processing hardware that mimics vision’s-spatial processing are described in McCann and Rizzi (2011) pp. (292–340). In all scene studied here, Contrast and Assimilation show distinctly different responses to light. Models of vision must predict both Illusions. Single pixels scene-independent models (sensor, film, Colorimetry) cannot predict either. Multi-resolution edge-detection techniques (Frankle and McCann, 1983; McCann and Rizzi, 2011) are needed to address Glare’s Paradox.

Please inspect the full-resolution retinal contrast patterns of five examples of Glare’s Paradox in Figure 12 in Data Sheet 7 in Supplementary material.

## Discussion

Since the 1960s, vision research and digital electronic imaging have produced an exponential growth in spatial-image-processing mechanisms. The work of Edwin Land, Fergus Campbell and John Robson, David Hubel and Torsten Wiesel, Gerald Westheimer and Suzanne McKee, Semir Zeki, Mark McCourt and Barbara Blakeslee expanded vision research by studying complex scenes. Instead of input pixels, they studied how entire scenes, or extended scene segments build appearances.

This article provides a new Python computer program that calculates the relative contrast of light imaged on the human retina. It also describes the analysis of scene_luminance input and retinal_contrast retinal response.

A previous study of glare, used HDR scenes with 1 million to 1 range (McCann and Vonikakis, 2018). The greater the range of luminances, the greater the magnitude of glare changes in the darkest regions. However, glare (on a pixel) is sum of all other scene pixels’ contributions. The content of the scene, and its local spatial arrangements of luminances generate unique glare patterns for every scene. This is because GSF does not approach a constant value. As shown in Figure 2 the CIE GSF maintains its high-slope decrease at 60° angular separation from the source pixel.

Contrast + Assimilation targets are the combination of lower-dynamic-range scenes (smaller glare magnitudes), and extreme “rest-of-the-scene” contents, limited to Whites and Blacks. The million-to-one HDR input range is reduced to 200:1 for these Illusions. This combination has a normal range of glare, and a large local glare re-distribution caused by max-and min-luminance scene content everywhere in the “rest-of-the-scene.”

Appearances are the consequence of glare plus neural processing. Glare is a simple optical process (rapid decrease in scatter with increase in visual angle). The GSF is convolved with all scene_luminances. All of the scene’s content is the co-creator of the spatial pattern of receptor responses.

### Visibility of gradients

Gradients are an essential sub-topic in vision. In the spatial-frequency domain, they live below the peak of the eye’s Modulation Transfer response function. Campbell and Robson (1968) transformed vision research in the 1960’s. They initiated decades of research in which oscilloscopes became vision research’s instrument of choice. Measurements of sinusoidal gratings at different frequencies generated vision models using Modulation Transfer Functions. Vision research moved from studying a few pixels to complex images and entire scenes. Campbell and Robson’s Contrast Sensitivity Curve was a plot of log Sensitivity (1/sinusoid’s detection threshold) for variable sinusoids (0.1 to 100 cycles per degree) with a peak at 3 c/degree and a lower slope decrease in sensitivity. The data reached a practical lower limit; at 0.1 c/degree one-cycle of sinewave target subtends 10°.

Land and McCann (1971) used gradient threshold to remove them from luminance input arrays in early Retinex Lightness models. McCann and colleagues measured the detection threshold of gradients.

*“At first, we thought that threshold was the range compression mechanism. It stimulated our MIT neighbors' interest in the problem. Tom Stockham described homomorphic filters, and Horn and Marr described Laplacian operators. These approaches applied mathematical functions to the removal of gradients. Our research at Polaroid turned in a different direction. If the threshold mimicked our human visual system, our model should have exactly the same properties as vision. We needed to measure the rate of change on the human retina that was at the threshold of detection. …We undertook a major effort to understand the visibility of gradients. We felt we needed better data on the rate of change of radiance on the retina that was at detection threshold to improve our model. It took 10 years, but we learned that there is no universal rate of change at threshold.”* (McCann and Rizzi, 2011*; p.312*)

McCann et al. (1974) measured the detection threshold of linear gradients at five different viewing distances (range = [4, to 16] feet, and [4.8°,to 1.2°] angle). Despite the 4× change in slopes of luminance gradients, detection thresholds were constant at all viewing distances. Savoy and McCann (1975) used threshold detection and supra-threshold matching to show that below the 3 cycle/degree peak, the visual detection thresholds for sinusoids no longer correlated with their spatial frequency. They found that the number of sinewave cycles correlated with visual responses. Hoekstra et al. (1974) found similar results. All that matters is angular size and number of cycles of sinusoid, and the size of the surround (McCann, 1978; McCann et al., 1978; Savoy, 1978; McCann and Hall, 1980; McCann, 2021b). Although we had proposed this rate-of-change threshold, we could not find psychophysical evidence for it as a visual mechanism. The Land and McCann gradient threshold, the Stockham spatial frequency filter, the Marr and Horn Laplacian can improve some pictures, but they do not have the same properties as vision. They cannot improve all pictures. Gradients are an under-appreciated special spatial challenge to vision research. As described above (Results), gradients are present in the retinal images, particularly in Lightness Illusions and real Natural Scenes.

### Glare’s role in image quality

Glare requires attention in quantitative image research. Glare adds a substantial modification of scene-content-dependent light on receptors. It is present in all accurate quantitative analysis of image data. We realize this every time we measure a scene with a well-designed low-glare-optics photometer, and compare its data with data from digital cameras [Camera digits ≠ Meter measurements] (McCann and Rizzi, 2007). Cameras capture scene radiances plus glare from camera’s optics. Cameras then add additional signal processing (McCann and Vonikakis, 2018). It is not possible to correct camera’s glare without knowing the data we are trying to measure (ISO 9358, 1994; McCann and Rizzi, 2011-pp.99–112). Glare’s scene-dependent re-distribution of light is difficult to observe (McCann, 2017). More important, glare redistributes the scene’s light in all scenes; it modifies both edges (higher-spatial frequencies) and uniform scene segments (lower-spatial frequencies).

### Neural spatial comparisons tend to cancel glare

Vision has two powerful spatial transforms of light from scenes: optical, then neural. Image quality of a scene_luminance array is degraded by optical veiling glare. However, receptor responses are the input to neural-spatial processing.

The central theme of Lightness Illusions is [Appearance ≠ scene luminance]. Contrast and Assimilation Illusions proved, a long time ago, that the “rest-of-the-scene” controls the appearance of scene segments. Many Lightness Illusions are designed with perfectly uniform segments (something that is rarely found in Natural Scenes). Uniform segments, with different luminances create a reasonable, but hidden assumption that these segments become an “object” with perceptual consequences. Glare upsets the “object” assumption. The uniform scene segments become a complex pattern of nonuniform light on receptors. After glare, populations of individual receptor responses cannot reliably report scene segmentation of “objects” to neurons. Sharp edges have become high-slope gradients. Other neural-spatial computations are needed to find and specify the location of objects’ edges that have become gradients (Figure 4).

All of the non-uniformities in Contrast + Assimilation experiments are not visible. All scene segments in these targets appear to be uniform patches on the computer display. Appearances are not accurate renditions of a receptor’s response to light. The lesson from Illusions is [Apparent Lightness ≠ scene luminance]. The lesson from this study is [Apparent “object” Uniformity ≠ retinal contrast and receptor responses].

Vision’s second spatial transformation is [Receptor responses ➜ ROI Appearance]. A comprehensive model of vision requires separate analysis of both independent transformations: optical and neural. Understanding appearances generated by scene_luminance is made more difficult because Glare’s Paradox shows these two strong spatial-transformations tend to cancel each other. All nine Lightness Illusions in this article contained pairs of GrayROI segments that showed Glare’s Paradox. Neural spatial processing not only cancels the effects of glare, it also overcompensates for it to create Glare’s Paradox. (In the 5 scenes that contained Assimilation Illusions, none of their pairs of GrayROI showed Glare’s Paradox.) Vision’s minimization of glare has the advantage that we rarely notice glare in everyday life. Neural-spatial comparisons, seen in Glare’s Paradox, overcompensate the effects of glare. Post-receptor-neural mechanisms emphasize edges, and minimize gradients.

Neural cancelation of glare creates a challenge for vision research; namely the separation of the independent optical effects from later neural effects. The psychophysical measurements of the neural effects caused by the “rest-of-the-scene” are severely underestimated when glare is assumed to be zero. In the Contrast experiments, the “Gray-in-White” has more light from glare. But, this “Gray-in-White” scene segment appears darker, showing Glare’s Paradox. The neural process compensates for glare’s increased luminance, and then overcompensates to make the “Gray-in-White” darker than the lower luminance “Gray-in-Black” segments. What we measure as psychophysical change in apparent lightness is a small residual difference from the sum of two-substantial lightness vectors in opposite directions. We need to know the glare-distorted receptor output to measure the magnitude of Contrast’s neural-spatial transformation in the opposite direction (McCann and Rizzi, 2011).

The combination of intraocular glare and Lightness Illusions shows complex-spatial-image-processing transformations following receptor responses. While optical veiling glare distorts the pattern of light from the scene, neural spatial processing cancels glare, and then over compensates for it. That is why glare is hard to see.

Instead of individual receptors, vision uses arrays of receptor responses to locate and synthesize sharp edges, and minimize the appearance of gradients. Post-receptor vision modifies the many local ranges of retinal_contrast to generate more useful appearances. Local neural-spatial processing is needed to compensate for the range of light in Natural HDR Scenes, and for glare in normal-range Lightness Illusions.

### Summary

This work adds essential facts to research in vision and image quality. Glare transformations of scene information are substantial in all of imaging, not just HDR.

1. While Lightness Illusion’s paradigm of equal stimuli holds in scene photometry, it fails for retinal receptor’s quanta catch and receptor responses.
2. Models of neural-spatial processing and human image quality must consider the actual spatial array of receptors’ quanta catch.
3. Nine examples of Glare’s Paradox shows that glare adds more light to GrayROIs with darker appearances; and less light to lighter ones. Neural spatial image processing cancels and then overcompensates the effects of optical glare.
4. Glare adds considerable light to Assimilation’s ROI that appear lighter. More research studies are needed to determine whether glare alone can predict Assimilation’s appearances. Both retinal receptor responses and appearances increase with increases in optical glare.

## Data availability statement

The original contributions presented in the study are included in the article/Supplementary material, further inquiries can be directed to the corresponding author.

## Ethics statement

Ethical review and approval was not required for the study on human participants in accordance with the local legislation and institutional requirements. Written informed consent for participation was not required for this study in accordance with the national legislation and the institutional requirements.

## Author contributions

JM and VV have collaborated on previous publication of MATLAB code for distribution. VV wrote and implemented the new code in open-source Python language and collaborated with JM and AR in the analysis. JM has brought together this glare and lightness research in collaboration with many others. All authors contributed to the article and approved the submitted version.

## Conflict of interest

The authors declare that the research was conducted in the absence of any commercial or financial relationships that could be construed as a potential conflict of interest.

## Publisher’s note

All claims expressed in this article are solely those of the authors and do not necessarily represent those of their affiliated organizations, or those of the publisher, the editors and the reviewers. Any product that may be evaluated in this article, or claim that may be made by its manufacturer, is not guaranteed or endorsed by the publisher.
